# Implementation of medicines pricing policies in sub-Saharan Africa: systematic review

**DOI:** 10.1186/s13643-022-02114-z

**Published:** 2022-12-01

**Authors:** Augustina Koduah, Leonard Baatiema, Anna Cronin de Chavez, Anthony Danso-Appiah, Irene A. Kretchy, Irene Akua Agyepong, Natalie King, Timothy Ensor, Tolib Mirzoev

**Affiliations:** 1 Department of Pharmacy Practice and Clinical Pharmacy, School of Pharmacy, College of Health Sciences, University of Ghana, Legon, Accra, Ghana; 2 Department of Health Policy, Planning & Management, School of Public Health, College of Health Sciences, University of Ghana, Legon, Accra, Ghana; 3 Department of Global Health and Development, London School of Hygiene and Tropical Medicine, London, UK; 4 Department of Epidemiology and Disease Control, School of Public Health, College of Health Sciences, University of Ghana, Legon, Accra, Ghana; 5 Ghana Health Service/Ghana College of Physicians and Surgeons, Accra, Ghana; 6 Leeds Institute of Health Sciences, University of Leeds, Leeds, UK; 7 Nuffield Centre for International Health, University of Leeds, Leeds, UK

**Keywords:** Policy implementation, Medicine pricing policies, Sub-Saharan Africa

## Abstract

**Background:**

High medicine prices contribute to increasing cost of healthcare worldwide. Many patients with limited resources in sub-Saharan Africa (SSA) are confronted with out-of-pocket charges, constraining their access to medicines. Different medicine pricing policies are implemented to improve affordability and availability; however, evidence on the experiences of implementations of these policies in SSA settings appears limited. Therefore, to bridge this knowledge gap, we reviewed published evidence and answered the question: what are the key determinants of implementation of medicines pricing policies in SSA countries?

**Methods:**

We identified policies and examined implementation processes, key actors involved, contextual influences on and impact of these policies. We searched five databases and grey literature; screening was done in two stages following clear inclusion criteria. A structured template guided the data extraction, and data analysis followed thematic narrative synthesis. The review followed best practices and reported using PRISMA guidelines.

**Results:**

Of the 5595 studies identified, 31 met the inclusion criteria. The results showed thirteen pricing policies were implemented across SSA between 2003 and 2020. These were in four domains: targeted public subsides, regulatory frameworks and direct price control, generic medicine policies and purchasing policies. Main actors involved were government, wholesalers, manufacturers, retailers, professional bodies, community members and private and public health facilities. Key contextual barriers to implementation were limited awareness about policies, lack of regulatory capacity and lack of price transparency in external reference pricing process. Key facilitators were favourable policy environment on essential medicines, strong political will and international support. Evidence on effectiveness of these policies on reducing prices of, and improving access to, medicines was mixed. Reductions in prices were reported occasionally, and implementation of medicine pricing policy sometimes led to improved availability and affordability to essential medicines.

**Conclusions:**

Implementation of medicine pricing policies in SSA shows some mixed evidence of improved availability and affordability to essential medicines. It is important to understand country-specific experiences, diversity of policy actors and contextual barriers and facilitators to policy implementation. Our study suggests three policy implications, for SSA and potentially other low-resource settings: avoiding a ‘one-size-fits-all’ approach, engaging both private and public sector policy actors in policy implementation and continuously monitoring implementation and effects of policies.

**Systematic review registration:**

PROSPERO CRD42020178166

**Supplementary Information:**

The online version contains supplementary material available at 10.1186/s13643-022-02114-z.

## Background

Over the past decade, the cost of accessing safe and quality healthcare has increased rapidly globally, attributed largely to the high prices of medicines [1]. Additionally, there are concerns that most of the highly priced medicines do not necessarily translate into improved health outcomes [2]. In response to high and increasing medicine prices, medicine pricing policies have been implemented to regulate prices of medicine and improve financial access [3, 4] to safe, quality and affordable medicines, one of the sustainable development goals in attaining universal health coverage by 2030 [5–7]. A medicine pricing policy can be defined as a set of written principles or requirements for managing the prices of medicines agreed or adopted by a public institution, a group of purchasing organizations or individual health services [8].

Various medicine pricing policies exist to regulate supply of essential medicines [8]. These policies can be categorized into (a) regulatory framework and direct price control, e.g. reference pricing, mark-up regulation, voluntary license agreement and tiered pricing; (b) targeted public subsidies, e.g. affordable medicines schemes; (c) generic medicine policy, e.g. promoting generic prescribing and use; and (d) purchasing policies, e.g. pooled procurement. Reference pricing is the practice of benchmarking or referencing a medicine price to the price in one or several countries or purchasing authorities [8]. Reference pricing remains a key policy widely employed globally as a regulatory policy [9–13]. Mark-up regulation represents the additional charges and cost applied to the price of a medicine along the supply chain, and this includes setting a single exit price at the ex-factory level [8]. Generic medicine policies are widely recommended and applied in many contexts [14–16] to influence medicine prices through competitions [8]. Pooled procurement through a single entity on behalf of individual purchasing authorities promotes competitive prices from manufacturers and suppliers [16–18].

The implementation of medicine pricing policies is influenced by multiple contextual barriers or facilitators of implementation approaches and processes. The facilitators include increased competition, skilful negotiations, pragmatic supply management and bulk purchasing [19]. Medicine pricing policies are challenged by the prevailing market conditions in a particular context, including proximity to particular medicines, quantities purchased and functionality of regulatory framework [20, 21]. There is, however, limited published evidence summarizing influences on the implementation of medicines pricing policies across low- and middle-income countries (LMICs).

Most medicine pricing policies have been implemented in high-income countries, but there is paucity of empirical data/evidence on implementation policies in LMICs, especially sub-Saharan Africa (SSA). Understanding medicine pricing policy implementation is particularly important as implementation of these policies can be a major challenge in LMICs where many patients with extremely limited resources need to provide out-of-pocket payments, thus impeding their access to medicines and putting them at further risk with increasing prices [11, 22]. Other studies focused only on the following: effects of reference pricing in organization for economic cooperation and development (OECD) countries [13], government initiatives to mandate drug pricing transparency [23], ensuring access to psychotropic medication [24], factors contributing to the increase in pharmaceutical expenditures [25] and generic drug policies in Brazil, Russia, India, China and South African (BRICS) countries [26].

With this backdrop, we conducted a systematic review on available medicine pricing policies in SSA, their implementation processes, contextual influences and impacts on prices and access to essential medicines. We addressed the main question: what are the key determinants of implementation of medicines pricing policies in sub-Saharan African countries? The review addressed four interrelated questions:

1. Which medicines pricing policies have been implemented in SSA and what are their key elements?
2. How have these policies been implemented (in relation to implementation approaches, processes, involvement of actors and their underpinning evidence)?
3. Which key facilitators and barriers affected implementation of medicines pricing policies and how?
4. What were the effects of medicines pricing policies with regard to reducing prices and improving access to medicines?

This review is aimed particularly at health policy analysts, healthcare professionals, implementation science scholars and decision-makers who are engaged in improving access to medicines in LMIC settings.

## Methodology

The review was conducted as part of a study on ‘Improving equitable access to essential medicines in Ghana through bridging the gaps in implementing medicines pricing policy, which involved collaboration between University of Ghana, Ghana Health Services and University of Leeds, with funding from the National Institute for Health Research (NIHR), UK’. The review follows the Preferred Reporting Items for Systematic Reviews and Meta-Analyses (PRISMA) Statement [27]. The review protocol was registered in the International Prospective Register of Systematic Reviews (PROSPERO, reference: CRD42020178166) and published [28].

### Search strategy

The literature search was initially run in April 2020 to identify studies covering implementation of medicine pricing policies in SSA. We updated and re-ran the searches on 25th May 2021 in Ovid MEDLINE(R) ALL 1946 to May 24, 2021, Embase (Ovid) 1996 to 2021 week 20; Global Health (Ovid) 1973 to 2021 week 20, Web of Science Core Collection, Scopus (Elsevier B.V) and African Index Medicus (via World Health Organization (WHO) Global Health Index Medicus). We also searched for grey literature in the Institutional Repository for Information Sharing (WHO) [29] and the World Bank Open Knowledge Repository [30]. For additional French-speaking articles, we searched the Erudit (University of Montreal) [31] and Cairn International (Cairn.info) databases [32].

Search strategies were developed using the major concepts: sub-Saharan African countries, medicine pricing, medicine policy and implementation. Database subject headings and free text words to search titles and abstracts were identified by the information specialist and project team members. The search terms and strategy were peer reviewed by a senior information specialist using the PRESS Checklist [33]. The searches were not limited by language but were restricted to studies published since 2000. This date was chosen following the introduction of the Millennium Development Goals (MDG) in 2000, which included a global focus on improving access to medicines and services. However, we did not search thee Department for International Development (DFID) or contact experts for additional papers as envisaged in the original protocol. Full search strategies are available in Additional file (see Additional file 1).

The results of the database searches were stored and de-duplicated in an EndNote X9 library. Further relevant studies were added by citation searching of the included studies from the following reviews [13, 23–26].

### Screening

A screening decision flowchart was agreed within the review team, which followed inclusion and exclusion criteria (see Additional file 2).

We included all empirical studies (randomized controlled trials (RCTs), quasi-experimental studies, cross-sectional and cohort studies) and reviews in English or French possessing the following criteria:

- A focus on medicine pricing policies to improve the affordability of medicines in the country
- A focus on how the policy processes were implemented
- A SSA context, published since 2000 with relevant information available for analysis

We excluded studies that were as follows:

- Opinion pieces, commentary or conceptual/theoretical publications
- Policy analyses which focused solely on the agenda-setting and development stages
- Conducted two plus years prior to 2000 but published after 2000 as this predates the MDG and Sustainable Development Goals (SDG) agenda
- In languages where we were unable to resource translation or where full text was unavailable

French articles were screened, translated and data extracted by native French speakers on the research team. Screening was conducted in two stages using the review management software Rayyan [34]. The first stage screening focused on the titles and abstracts and the second on full texts. To ensure consistency across the team, the initial titles and abstracts of 50 records were independently screened by eight researchers, and the results were discussed to reach consensus and standardize approach and calibration. The remaining records were then randomly allocated (295 each) for independent screening. One researcher (T.M) screened the remaining records and co-screened 20% of the records from each reviewers’ subset for consistency. In the second stage, full-text articles were independently reviewed by two researchers (A. C, L. B) against the inclusion criteria. Discrepancies were resolved in discussion between these members and with involvement of two further reviewers (A. K. & T. M.).

### Quality assessment

Quality assessment on the included studies was performed independently by two researchers (A. C., L. B.) using the relevant critical appraisal checklists from the Joanna Briggs Institute to assess the methodological quality of the eligible studies. Where discrepancies existed, two other reviewers were consulted (A. K., T. M.). Three quality assessment tools for cross-sectional analytical studies, cohort studies and qualitative studies from the Joanna Briggs Institute tools were used. For the analytical cross-sectional quality assessment, eight domains were assessed as ‘Yes (present), No (absent), Unclear (insufficient information) or Not Applicable’ [35]. The checklist for the qualitative studies has 10 domains assessed as ‘Yes (present), No (absent), Unclear (insufficient information) or Not Applicable’ [36]. Similarly, the checklist for the cohort studies has 11 items and is assessed as *yes (present)*, *no (absent)*, *unclear (insufficient information)* or *not applicable* [37]*.* The checklist criteria were not modified but interpreted flexibly to reflect our focus on the implementation of medicines pricing policies. As a result, the overall scores or results were presented narratively to reflect the presence (yes) or otherwise (no) of which of the domains.

### Data extraction and coding

Two authors (A. C., L. B.) extracted the data from all the 31 studies using a Microsoft Word template. The forms were designed to include publication details (author, date, country, study design; date study conducted); medicines pricing policy (key elements, effects on prices, effects on healthcare access); the policy implementation approach (processes, actors, evidence use); and any facilitators and barriers to policy implementation and their effects. Extracted data were coded in identifying the main themes emerging as shown in Table 1.

**Table 1:** Themes and subthemes

Themes	Subthemes
Medicines pricing policies implemented	Targeted public subsides
	Regulatory framework
	Generic medicines policies
	Purchasing policies
Policy implementation approach	Use of private distributors
	Regulatory framework
Use of evidence in the policy implementation design	Type of evidence used
Actors involved in policy implementation	Government
	Retailers
	Wholesalers
	Manufacturers
	Professional bodies
	Donor agencies
	Public and private health facilities
Barriers to policy implementation	Contextual factors serving as barriers at micro level (i.e. individual/personal)
	Contextual factors serving as barriers at meso level (i.e. organizational)
	Contextual factors serving as barriers at macro level (i.e. national systems)
Facilitators to policy implementation	Contextual factors serving as facilitators at micro level (i.e. individual/personal)
	Contextual factors serving as facilitators at meso level (i.e. organizational)
	Contextual factors serving as facilitators at macro level (i.e. national systems)
Effectiveness of implemented policies	Control or reduce medicine price
	Improve access to healthcare, i.e. availability and affordability

### Data analysis and synthesis

Due to the heterogeneous nature of medicines pricing policies, and the countries involved, we conducted a thematic narrative synthesis of the data [38], which followed the four review questions. The thematic summaries in Table 1 were developed drawing on the review questions to categorize the study findings into thematic groups [38]. The findings were synthesized, organized and reported around the main themes and subthemes.

## Results

**Fig. 1:**
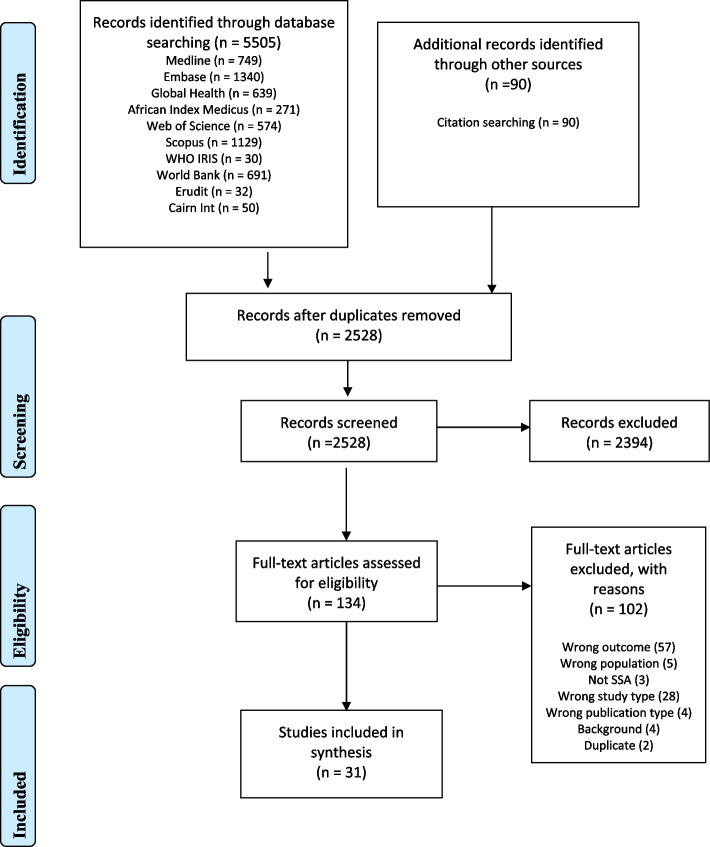
PRISMA flow diagram to illustrate the screening process from the initial search until the final selected studies (Page et al., 2021)

The final searches identified 5505 records, and citation searches identified a further 90 records. Once duplicates were removed, there were 2528 records. Screening by titles and abstracts identified 134 records for full-text review, and 31 studies were eligible for inclusion, data extraction and analysis. Studies were excluded based on wrong outcome (*n* = 57) because the intervention of interest was not present, population (*n* = 5), study type (*n* = 28) such as commentary and theoretical publication, publication type (*n* = 4) and background article (4) or duplicate (2), not SSA (*n* = 3), and these are detailed in the PRISMA flow diagram (Fig. 1). The eligible studies were drawn from multiple study designs but mostly quantitative nature, cross-sectional in nature (e.g. [39–44] and retrospective studies (e.g. [45–47]). The characteristics of studies included in the review are listed in Table 2.

**Table 2:** Characteristics of the studies included in the review sample size, study design and settings

Citation	Geographic focus	Objective/study description	Study design/type	Study population and sample size	Medicine pricing policy studied	Implementation of medicine pricing policies			Key influencers on implementation		Policy effects	
						Approaches	Actors	Use of evidence	Facilitators	Barriers	Effect on prices of medicines	Effect on access to essential medicines
Ali and Yahia, 2012 [36]	Sudan	To compare national pricing with retail prices, adherence of prices, comparison of generic medicine pricing, demonstrate violations and put forward recommendations	Cross-sectional survey	Survey of 174 medicines	Pharmacy and Poisons Act (2001)	Regulation of pharmaceutical prices using the current fixed mark-ups of 15% and 20% of the total cost for wholesalers and retailers, respectively	Wholesalers, National Medicines and Poisons Board, manufacturers and retailers	N/A	N/A	-Shortage of trained personnel and resources to assess cost and freight (C&F) prices -Lack of scrutiny on medicine pricing information by regulators -No pricing control, e.g. medicine prices of certain generics higher than their originators	-23% of C&F prices approved by NMPB were over 10 times the international reference price -The wholesale and retail prices were 40% and 47% less than that approved by NMPB respectively -E leven out of 12 originator medicines were ≥ their retail prices published in the British National Formulary -Prices distributed by Central Medical Supplies was 2-fold their C&F price	N/A
Ali, 2009 [48]	Sudan	To evaluate the revolving drug fund (RDF) effect on accessibility of essential medicines and its impact on the utilization of public health services	Mixed methods: structured interviews and documentary analysis	Ninety-three patients at the exit points of selected public health facilities. Ninety-three health facility users in hospitals and health centres, 5 with the RDF and 2 without. One teaching hospital outpatient department, 1 rural hospital and 3 health centres (urban, peri urban and rural)	Revolving drug fund	N/A	Ministry of Health Save the Children (UK)	N/A	Initial funding support from donors	N/A	-Cost of RDF prescription was perceived as affordable by users with improved quality compared to previously free medicines -Medicines for chronic diseases were considered expensive -Patients in non-RDF facilities spent more than those in RDF facilities, with 67% able to afford their medicines and 8% discontinued due to cost	RDF facilities had increased access to essential medicines, with 97% availability during the past 12 months
Ashigbie et al., 2016 [49]	Ghana	To examine medicines managements policies under Ghana’s NHIS, from perspectives of public and private sector providers	Qualitative study using semi-structured interviews	Public and private sector providers (government and mission hospitals, private hospitals and private standalone pharmacies), pharmaceutical suppliers and NHIS district office	Reimbursement of medicines to health facilities	Reimbursement for cost to private and public health facilities	-Public and private health facilities -Pharmacies -Licenced chemical shops -Christian Health Association of Ghana Facilities	N/A	An essential medicines list facilitates reclaim costs of a wide range of medicines	-Lack of standardization of mark ups (25–50%) and high market prices of medicines -Reimbursement delays	-Lower prices at CMS does not apply in pricing in retail pharmacy -The current pricing system, in both public and private sectors, is of limited benefit in controlling escalating medicine prices	Patients may not have access to medicine because not all facilities participate in the scheme and not all medicines are captured in the NHIS reimbursable list
Bangalee and Suleman, 2016 [45]	South Africa	To examine cardiovascular originator and generic drug prices using international reference prices	Quantitative study — secondary data analysis	Five classes of cardiovascular disease drugs	Generics and single exit price (SEP) legislation	Manufacturers could sell their medicines at uniform prices	N/A	N/A	Prices lowered based on market availability	N/A	-The SEP policy has not resulted in competitive prices -75% of generic drugs were 40% or more cheaper than the branded ones	N/A
Bangalee and Suleman, 2019 [46]	South Africa	To compare prices among originator, pseudo-generics and generics	Quantitative study based on private sector prices of medicines	Prices taken from 18 medicines in study	Generic medicines and SEP	SEP mandates manufacturers to sell at a uniform price	N/A	N/A	N/A	-Lack of prices regulation -Established relationship for originator companies creating challenges for generic manufacturers	N/A	N/A
Cassar and Suleman, 2019 [50]	South Africa	To assess whether international benchmarking of medicines (IBM) with comparator countries would lower medicine prices locally	Quantitative economic — observational analysis	Not documented	External reference pricing (ERP) [39] policy	SEP aimed to regulate, pricing, remove rebates and discounts	-Pricing committee -National Department of Health	N/A	N/A	-The use of ERP does not adopt a multidimensional approach -Emergence of negotiated confidential discounts	Ex-manufacturer price reduced by 68%, 85% and 85% of products in 2016, 2017 and 2018, respectively	N/A
Cohen et al. 2013 [51]	Tanzania	To assess the first 1.5 years of Affordable Medicines Facility for malaria (AMFm) use in Tanzania	Household longitudinal survey and interview, surveys and customer exit interviews	Sixty-four ADDO shop owners. Sixty-four sub villages, Seven-hundred households in round 1 and 756 in rounds 2 and 3. Total = 3900 individuals	N/A	N/A	N/A	N/A	Awareness campaigns	People not being aware that artemisinin combination therapies (ACTs) were a better treatment option	N/A	ACT use from round 1 to 3 increased
d'Almeida, et al. 2011 [47]	Cameroon	The study presents lessons learnt from provisions of second-line treatments for HIV and AIDS	Mixed methods — interview; semi-open questionnaires	Not stated	Free medicines	Free second-line treatments for HIV/AIDs	National Council for the Fight Against HIV/AIDS, National Direction to Fight Diseases, Provincial Centers for Treatment	N/A	Free second-line treatment facilitated by external funding	-Lack of integrated information systems on HIV/AIDS patients -Deficiencies in the supply chain/logistics for 2nd-line treatments	N/A	Problems led to very limited number of patients getting 2nd-line treatments
de Jager and Suleman, 2019 [44]	South Africa	To determine the impact of generics and generic reference pricing on candesartan and rosuvastatin	Quantitative, retrospective	Beneficiaries from registered medical schemes who were contracted with the PBM for the entire study period	Generics and reference pricing	N/A	Government, pricing committee, pharmacists, Pharmaceutical Society of South Africa and Retailers	N/A	N/A	A small number of generics manufacturers in South Africa	Average price reductions range from 13.9 to 31.0% for rosuvastatin and candesartan, respectively	Utilization of rosuvastatin increased from 24.0 to 63.9% and then 76.4% following the introduction of the generic reference pricing
Fink, et al. 2014 [41]	Uganda	To determine the effect of AMFm on the use of ACTs	Cross-sectional survey-baseline survey	Targeted retail outlets including small, informal, unlicensed shops and vendors to licensed pharmacies	Affordable Medicines Facility for malaria	N/A	-Global fund -UNITAID -Gates Foundation	N/A	N/A	-Public sector stock outs, high prices in drug shops and pharmacies -Limited geographic coverage	AMFm benchmark was achieved even prior to the arrival of the program and sustained throughout	-ACT increased from 51 to 68% -More shops stocked ACTs, leading to 52% AMFm
Guimier et al. 2005 [52]	Senegal	To highlight differences between the price of drugs in Senegal and the population’s ability to pay for them		Wholesale distributors and 532 private pharmacies operating through a network of pharmaceutical depots and the National Supply Pharmacy	Reimbursement policy	Reimbursement of medicines in the public sector	Private and public pharmacies, wholesalers, manufacturers, laboratories, distributors	N/A	N/A	N/A	-The components of the public price vary only slightly between the four categories of medicines: taxes (1.3–1.4%), freight, insurance and local transit (5–6%), distribution margins (40–48%) and PGHT (46–54%)	Only 5% of patients had not taken their prescribed drugs for financial reasons
Honda and Hanson, 2013 [53]	Madagascar	To assess the outcomes of the equity funds in Madagascar from three perspectives	Household survey	Households. Three case studies Case 1 — urban (all amenities) Case 2 urban/rural (suburban) Case 3 rural (few basic amenities)	Pooled procurement & user fees: equity fund	Community participatory approach	Government and Community representatives	N/A	Knowledge of implementation status	Financial and geographical constraints accessing health centre	Out-of-pocket payments lower for members than non-members	Equity fund members have increased access to the public health facility
Liu and Galárraga 2017 [54]	Angola, Botswana, DRC, Lesotho, Malawi, Zambia Mozambique, Namibia, South Africa, Swaziland Tanzania and Zimbabwe	This study aims to (i) analyse global ARV prices from 2004 to 2013 and (ii) examine the relationship of national drug policies to ARV prices	Price survey Descriptive drug price trends 2004–2013	No participants	-Essential medicines list -National or social health insurance -Procurement strategy	N/A	N/A	N/A	-Transaction volume -HIV prevalence	N/A	-Generic status 8/10 ARVs had lower prices than originat -All six first-line ARV drug unit prices decreased over time, from a 46% price decrease for lamivudine to 90% price decrease for efavirenz	N/A
Maiga, et al., 2010 [55]	Mali	To analyse the role of government intervention and market forces in price regulation, private sector pricing of essential medicines and pricing process in Mali’s private pharmaceutical sector	Qualitative study	Not documented	Government price regulation policy	Set up a commission, price ceilings, monitoring and evaluation system and define working methodology for access to medicines	Managers, pharmaceutical companies, employers’ council, union workers and pharmacy professionals	N/A	High involvement of private and public sector stakeholders	Disagreement between the public and private sector	Estimated 25% theoretical reduction on the basket of 107 medicine	N/A
Maïga, and Williams-Jones 2010 [40]	Mali	To assess the impact of the national pharmaceutical policy on supply system for generic essential medicines	Price survey, a cross-sectional descriptive survey	Sixteen wholesalers and 30 private drugstores	Generic essential medicines	N/A	-Government -Private and public healthcare sectors	N/A	Education and creating awareness	N/A	The median wholesale price of the 49 drugs was 14.3% and 25.6% cheaper than the maximum price in 2006 and 2009, respectively	The availability was judged to be the same before and after the policy
Maïga, et al., 2003 [56]	Mali	To study cost recovery and generics policies	Price survey and observations of customers	Pharmacies and public health centres	Cost recovery and generics	N/A	N/A	N/A	N/A	N/A	Costs of prescriptions were lower where public health facilities had been revitalised	Access to drugs was improved affordable generics were widely available, even in private outlets
Moodley, R. and Suleman, F., 2019 [57]	South Africa	To evaluate the impact of SEP on a basket of originator medicines, in terms of costs, and impact on prices	Longitudinal before and after evaluation study	No participants — data used ‘The Global Core of fourteen items (14) originator and forty-six (46) generics	Single exit price policy	N/A	N/A	N/A	N/A	N/A	Upon introduction of the intervention, the medicines showed an immediate drop in price with a subsequent rate of increase being much less than before	N/A
Moodley, R. and Suleman, F., 2019 [58]	South Africa	To examine the impact of the regulatory change, the SEP, on a basket of generic medicines from 1999 to 2014	Quantitative study	Prices of 50 originator medicines were assessed from 1999 to 2014	Single exit price policy on generics prices	N/A	-Manufacturers -Pricing committee -Ministry of health	N/A	N/A	N/A	The SEP had a larger effect on generics pricing than originator. Most medicines showed a smaller yearly increase in price compared to before regulations	N/A
Nicolosi, E. and Gray, A., 2009 [59]	South Africa	To assess the potential savings by substituting generics for brand	Economic evaluation study	All the medicines listed in 25 chronic disease algorithm made by the Council for Medical Scheme	Generic medicines policy	N/A	N/A	N/A	N/A	N/A	67.5% were more than 40% cheaper than branded medicines. All generics were priced lower	N/A
Ongarora, et al. 2019 [60]	Kenya	To assess retail pricing, availability and affordability of medicines in private facilities	Survey using standardized electronic questionnaire	Forty-five private healthcare facilities in 14 of Nairobi’s low-income settlements (18 clinics, 7 hospitals, 2 health centres, 4 medical centres, 2 nursing and maternity homes, 12 pharmacies) 28 innovator products were included	Generic medicines Policy	N/A	N/A	N/A	N/A	The lack of regulation of prices	Clients paid higher prices than the median IRPs for 68.6% of generic medicines selected	N/A
Ponsar, et al., 2011 [39]	Mali	To assess the impact of abolishing user fees on utilization of essential health services and mortality	Survey	Pregnant women and children under five	Subsidized/free medicines for malaria treatment	N/A	-MSF (doctors without borders) -Health centres -Ministry of Health	N/A	Free provision medicines	Payment of user fees	Savings in drugs reduced the overall consultations cost	Utilisation of healthcare increased fourfold for under 5 s; by the end of the period, 3.5 × more pregnant women were being treated for fever
Rothberg, et al. 2004 [61]	South Africa	To measure the impact of reference-pricing programme covering items for available generic equivalents	Prospective and retrospective analyses of prices of medicines Quantitative — price survey	All medicines for which generics products were available	Reference pricing for generic medicines	N/A	-Medscheme’s medicines management -Interpharm teams -Government	N/A	Willingness of some manufacturers to drop prices	Low enrolment into the programme	Price movement for eligible products for the 12-month period showed that 19.6% of products dropped prices, 16.8% increased by up to 10%, 19.5% by 11 — 15%, 7.8% by 16 — 50%, 1.7% up to 100% and 1.0% by more than 100%	N/A
Sabot, et al. 2009 [38]	Tanzania	To evaluate the extent to which patients use recommended ACTs and its implications for AMFm implementation	Cross-sectional study — exit interviews, retail audits, mystery shoppers, and public facility audits	Drug shop customers, retail audits	Affordable Medicines Facility-malaria	N/A	Wholesalers and retailers	N/A	-Popularity of designated retail outlets -Global policy and funding	-Cost is still a barrier for poorer customers -Stock-outs and challenges with the supply chain	Consumers purchasing ACTs for children under 5 paid significantly less than those buying for adults	Increase in the proportion of shops stocking ACTs in the intervention districts, from 0/133 in August 2007 to 109/151 (72.2%) in August 2008
Smith, et al. 2011 [37]	Kenya	To measure accessibility, availability and affordability of ACT	Survey	All public health facilities and malaria medicine retailers, including private clinics, chemists, pharmacies and other specialized drug stores	Affordable Medicines Facility-malaria	N/A	Government Global fund	N/A	-Proximity to and flexible business hours of retail facilities	-Most of the drug outlets were unlicensed -Frequent stock-outs in public facilities	Brands purchased under the AMFm programme cost 40% less than non-AMFm brands	Increased access for those buying drugs at weekends from private outlets
Steyn, et al. 2007 [43]	South Africa	To determine the influence of implementing SEP on the prescribing prevalence and cost of antidiabetic medicine	A retrospective drug utilisation study conducted in 2005 and/or 2006	Private sector healthcare	Reference-based pricing system (single exit price).	N/A	-Manufacturers -Wholesalers -Retailers -Government	N/A	1997 Medicine and Related Substances Amendment Act	N/A	The average cost of antidiabetic medicine on the database decreased from the pre-SEP period and interim period in the post-SEP period	Prescribing frequency of antidiabetic medicine showed an increase
Tougher, et al. 2014 [62]	Ghana, Kenya, Madagascar, Niger, Nigeria, Tanzania	To examine the potential for further reductions in the prices of subsidized medicines	Quantitative, price survey	Retail outlets	Affordable Medicines Facility -malaria (AMFm)	N/A	N/A	N/A	-Already existing ACT subsidy policy -Accessibility of private retail facilities	Lack of standardized mark-ups for retail pharmacy	Prices reduced in most countries	N/A
Tran et al. 2020 [42]	Kenya	To describe how the evolution of the RFP programme increased access to essential CVD medications for patients across different levels of the public sector healthcare system in western Kenya	Retrospective study using administrative data	Inventory audit reports, essential CVD medicines list	Revolving fund pharmacy model	Donations or purchase sold at a small mark-up price sufficient to replenish drug stock and ensure sustainability	Kenya MOH Health facilities [community), level 2 (health dispensaries), level 3 (health centres), level 4 (subcounty hospitals), level 5 (county hospitals), to level 6 (tertiary referral hospitals)]	N/A	Kenya MOH, local leadership and facility administrators’ effort to integrate CVD and diabetes clinical services as well as essential medications into the lower primary care-level facilities Creation of local adoption mechanisms Early engagement of key stakeholders Developing affordable patient co-pays, waivers and accountability mechanisms through inventory, financial and accounting systems	Transportation costs to health facilities, opportunity cost of missed work and distance from health facilities Significant operating costs associated with running the pharmacies including staff, co-pay waivers, supervisory audits and transportation of medicines and supervisors Patient volumes at each of these lower-level facilities were not sufficient to sustain a full RFP Clinical officers or nurses were too overwhelmed to dispense and maintain the inventory of RFP medicines	N/A	The availability of essential medicines improved from an average of 30–40% to > 90%, 18. In the period of the current analysis (2018), this model was run in 15 facilities within the AMPATH catchment area Most tracer medicines were present 94–100% of the time at health facilities across levels 2–6 (the availability of insulin (Humulin 70/30) at levels 5 and 6 was 97% and 100%, respectively, and 81–85% at levels 2–4) An increase in the availability of generic CVD medications from the historical 30% or less to 90% or higher across all levels of the health system
Walwyn and Nkolele, 2018 [63]	South Africa	To evaluate whether private-public partnership (PPP) of the Biovac Institute provided value for money for vaccine procurement and distribution over the period 2010–2016	Concurrent mixed methods	Quantiative — prices from secondary sources Qualitative — ‘key stakeholders’ representatives from BI, National Treasury, National Department of Health (NDoH), provincial departments of health, the Technology Innovation Agency, the Industrial Development Corporation, the Department of Science and Technology and the Department of Trade and Industry were invited; of these, 5 agreed to be interviewed	Public-private partnership (PPP) policy for vaccine procurement and distribution	N/A	National Treasury, Department of Health, Technology Innovation Agency, Industrial Development Corporation, Departments of Science & Technology and Trade & Industry	N/A	-Uninterrupted/reliable supply chain -Political support for PPP	Slow establishment of a vaccine manufacturing centre Forex fluctuation (depreciation of the local currency	Biovac Institute has been successful in containing the cost of procurement for the EPI vaccines, and that this competence has been strengthened over the period of this study The margin averaged at approximately 13%, corresponding to a total value of US $85.7 million over the period of the evaluation or about US $17million per year	No interruption in the supply of vaccines to any location in the country
Wiedenmayer, 2019 [64]	Tanzania	To develop a successful pilot of a prime vendor system with the potential for national scale-up.	Baseline survey and M&E reports	National Coordination Committee was formed, composed of members from ministries and agencies. Regional and district stakeholders and health care workers	Jazia prime vendor system (public-private partnership)	Engaging one private sector pharmaceutical supplier as the prime vendor to provide the complementary medicines needed by public health facilities in Tanzania	-Private sector -Government -Medical stores department -Health facilities -National Coordination Committee	N/A	-Partnership with private sector -Culture of transparency and accountability -Regional leadership	Delayed payment by the districts for their PV consignments (up to 90 days)	N/A	Tracer medicines availability in the region (mean availability of all districts) increased from 69% in 2014 to 94% in 2018
Wilson, 2012 [65]	Tanzania	To assess the manufacturing capacity to produce ARVs locally	Mixed-methods case study: quantitative data from document review, qualitative data from semi-structured interviews and document review	Representatives from government agencies, the pharmaceutical industry and international, bilateral organizations and NGOs	Generics and domestic production policy (TRIPS and Doha Declaration)	N/A	Tanzania Pharmaceutical Industries Government	N/A	Existing international polices supporting domestic production of drugs	-Lack of a coherent policy strategy for the development of its pharmaceuticals industry -Weak patent enforcement -High costs of importing supplies	N/A	N/A
Ye, 2015 [66]	Ghana and Kenya	To assess the availability, price and market share of quality-assured artemisinin-based combination therapy in remote areas compared with non-remote areas at end line of the AMFm intervention	Cross-sectional	Data collected from drug outlets in Kenya and Ghana	Affordable Medicines Facility -malaria	N/A	-Government -Global fund	N/A	-Available funding to subsidize the drugs on a global level -Reliable distribution systems -Community awareness	Remoteness of private outlets	-In Ghana, the prices in remote and non-remote areas did not differ public health facilities -In Kenya, private for-profit outlets in remote areas were selling QAACT at nearly twice the price as in non-remote areas	Medicines were available in both Kenya and Ghana

### Quality assessment

Results of the quality assessment are presented in Additional file (see Additional file 3). For cross-sectional analytical studies, a total of 22 out of the 23 studies reported a clearly defined inclusion criteria for the recruitment of participants and description of outcomes to be considered for the study. All the eligible studies provided sufficient information about the study participants and settings. However, only six studies provided information to indicate how potential confounding factors were identified or accounted for, and similarly, little information existed on how confounding factors were addressed. For the qualitative studies, only one study reported information on the philosophical perspective, making it difficult to establish congruity with the research objectives and methodological approaches adopted. However, congruity was established between the research methodology and the data collection methods, analysis and interpretation of the results. None of the studies also reported how the researcher could have potentially influenced the research process. Lastly, for the cohort studies, the nature of the studies did not permit quality appraisal of the three included studies due to limited information. For example, little or no information was provided on how potential confounding factors were identified and dealt with to minimize bias. There was information on the follow-up period in one out of the 3 studies reported. Follow-up was completed for only one study, and strategies to address incomplete follow-ups were not utilized. Measurement of exposures was not done uniformly across both exposed and unexposed populations; thus, the risk of bias was unclear. Statistical analyses adopted in the studies were relevant and reported results addressing the study objectives. Overall, on the cohort studies, there was limited information to sufficiently appraise the studies, thus further increasing ambiguity and risk of bias of the included studies.

### Medicine pricing policies implemented in sub-Saharan Africa

In this section, we report results based on the four review questions.

#### Types of medicine pricing policies implemented in SSA

The 31 articles identified in the review revealed a total of 13 medicine pricing policies were implemented across SSA countries between 2003 and 2020. These policies represent four domains, shown in Table 3: (1) targeted public subsidies, (2) regulatory framework and direct price control, (3) generic medicine policies and (4) purchasing policies.

**Table 3:** Distribution of medicine pricing policies according to implementation countries

Domains	Specific medicine pricing policy	Country	Reference
**Targeted public subsidies**	Affordable Medicines Facility for malaria	Uganda, Tanzania, Kenya, Ghana, Nigeria, Niger, Madagascar	Fink, 2014 [41], Sabot 2009 [38], Smith, 2011 [37], Tougher 2014 [62], Ye 2015 [66]
	Free medicines scheme	Cameroon, Mali	d’ Almeida 2011 [47], Ponsar 2011 [39]
	Equity fund	Madagascar	Honda 2013 [53]
	Subsidy schemes	Tanzania, Uganda, Senegal	Ponsar 2011 [39] & Tougher 2014 [62]
**Regulatory framework and direct price control**	State price regulation frameworks	Angola, Botswana, Democratic Republic of Congo, Lesotho, Malawi, Mozambique, Namibia, South Africa, Swaziland, Mali, Tanzania, Zambia and Zimbabwe	Liu 2017 [54], Maiga 2010 [55]
	Reference-based pricing systems	South Africa	Casar & Suleman 2019 [50], de Jager & Suleman 2019 [44], Rothberg 2004 [61], Steyn 2007 [43]
	Single exit price (SEP) policies	South Africa	Steyn 2007 [43], Moodley & Suleman 2019a [57] and Moodley and Suleman 2019b [58], Bangalee and Suleman 2016 [45], Bangalee and Suleman 2019 [46]
	Pharmacy and Poisons Act	Sudan	Ali and Yahia 2012 [36]
	Reimbursement schemes	Ghana	Ashigbie 2016 [49]
**Generic medicine pricing policies**	Generic medicine pricing policies	South Africa, Mali, Kenya	Bangalee and Suleman 2016 [45], Bangalee and Suleman 2019 [46], de Jager and Suleman 2019 [44], Maïga 2010 [55], Nicolosi 2009 [59], Ongarora 2019 [60] and Wilson 2012 [65]
	Cost recovery and generics	Mali	Maïga 2003 [56]
**Purchasing policies**	Public-private partnership	South Africa, Tanzania	Walwyn 2018 [63], Wiedenmayer 2019 [64]
	Revolving drug fund policy	Sudan, Kenya	Ali, 2009 [48] and Tran et al. 2020 [42]

**Fig. 2:**
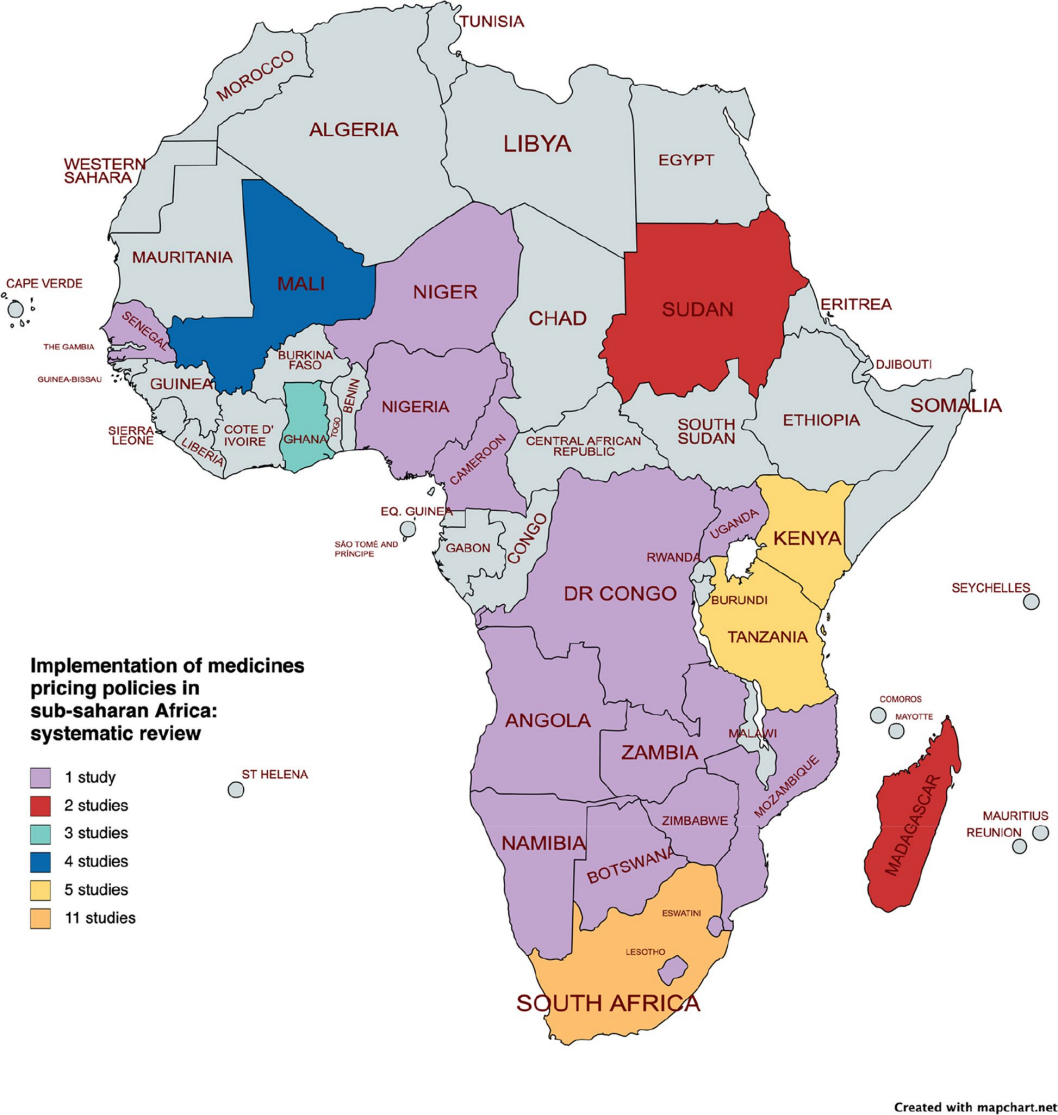
Distribution of selected studies per countries in SSA

Medicine pricing policies were reported from 22/46 countries in SSA, with 11 of the 31 studies reported from South Africa [46, 50, 54, 57–59, 61–64, 66]. As shown in Fig. 2, most were single-country studies, but a few were based on a multicountry data [51, 55, 59].

Some studies focused exclusively on evaluating effects of a medicine pricing policy on access to healthcare [48, 58, 60], the effect of the policy on prevailing prices of medicines [39, 50, 52–55, 59, 61, 62, 66]. Others, however, evaluated policy effects on medicine prices and access to medicines [40–44, 46, 47, 49, 51, 56, 64, 65, 67].

#### How medicine pricing policies were implemented?

##### Approaches to policy implementation

Approaches to policy implementation were described in 10/31 studies. Two main implementation approaches for medicine pricing policies were evident in the data: (a) use of private distribution outlets and networks and (b) use of government’s regulatory frameworks. Three studies [56, 58, 60] were described using private distribution outlets and networks to improve financial and geographical access of medicines through pooled procurement and subsided schemes. For example, in Tanzania, the government engaged private sector pharmaceutical supplier as the prime vendor to provide complementary medicines needed by public health facilities [60].

The second approach involved the use of regulatory frameworks to guide the sale and distribution of medicines [39, 52, 54, 57, 62, 65, 67]. Seven reported on the use of regulatory frameworks. Reduction in reference price was an approach used to set price ceilings for a particular group or category of medicines including generic medicines [62]. The implementation of the single exit price (a fixed ex-factory price) policy was also observed in different contexts [46, 50, 54, 57, 61].

##### Actors in policy implementation

A total of 22/31 studies highlighted seven groups of policy actors who were involved in policy implementation. These comprised the following: g*overnment* [40, 42, 43, 46, 47, 49, 51, 56, 58, 60–62, 66, 68], *wholesalers* [39, 41, 46, 65], *retailers* [39, 41, 46, 47], *manufacturers* [39, 46, 50, 65, 68], *donor agencies* [40, 42, 44, 49, 51], *professional bodies* [47, 52], *community members* [56] and *public and private health facilities* [43, 52, 60, 65, 67]. Information reported in the studies covered largely actors’ roles in policy implementation, but did not report actors’ interests, agendas and relative powers.

##### Evidence to inform policy implementation

None of the studies reported on the use of evidence to inform implementation as well as monitoring and evaluating implemented policies, although few studies (2/31) explicitly described evidence use in informing the design of medicine pricing policies. Where it was reported, evidence was sourced from review of pharmaceutical pricing policies [48] and a WHO report on medicine access and procurement of medical commodities [64].

#### Key facilitators and barriers to implementation of medicine pricing policies

Multiple contextual facilitators and barriers to the implementation of the medicine pricing policies in sub-Saharan Africa were identified in this review. These were across the micro (individual), meso (organizational) and macro (national) levels (Table 4). Five studies only reported facilitators [43, 46, 49, 54, 59], six reported only barriers [39, 44, 47, 53, 57, 62] and 14 reported both barriers and facilitators [40–43, 45, 48, 51, 55, 56, 58, 60, 64, 66–68].

**Table 4:** Key facilitators and barriers to implementation of medicines pricing policies

	Facilitators	Barriers
**Micro level factors**	Knowledge of implementation status (Honda 2013, Cohen 2013 and Maiga 2010) [51, 53, 55]	Long-distance travel by individuals (Ye 2015, Tran et al. 2020) [42, 66]
	Village with a drug shop (Smith 2011) [37]	
	Alternative drugs that are less effective but cheaper preferred by public (Cohen 2013) [51]	
**Meso level factors**	Drug and therapeutic committee to regulate prices at the facility level (Ashigbie 2016) [49]	Limited access to medicines, frequent stock outs (Fink 2014, Honda 2013, Ye 2015, Smith 2011) [37, 41, 53, 66]
	Pooling resources and buying in bulk (Ashigbie 2016) [49]	Shortage of trained personnel and lack of resources to scrutinize prices of medicines and information about medicine prices by the pharmaceutical companies (Ali and Yahia 2012, Tran et al. 2020) [36, 42]
	Lower prices increase access (Cohen 2013) [51]	Lack transparency of prices in an external reference pricing (ERP) comparison where confidential discounts are negotiated (Cassar & Suleman 2019) [50]
	Subsidies/free provision of medicines (Ponsar 2011) [39]	Lack of printed retail prices on medicine pack (Ali and Yahia 2012) [36]
**Macro level factors**	Existing national medicine pricing policies (Steyn 2007, Ashigbie 2016) [43, 49]	Lack of state capacity to regulate (Ali and Yahia 2012) [36]
	Strong political will from government (Walwyn & Nkolele 2018, Wiedenmayer 2019, Tran et al. 2020) [42, 63, 64]	Delays in reimbursement of health facilities and supplies (Ashigbie 2016) [49]
	Donor agencies and international policies and interventions (Wilson 2012, Ye 2015, Sabot 2009, Ali 2009) [38, 48, 65, 66]	Lack of scrutiny on medicine pricing policy by regulators (Ali and Yahia 2012) [36]
		Lack of a coherent and well-functioning national medicine pricing policy (Wilson 2012) [65]
	Use of essential medicines list (Ashigbie 2016) [49], Medicine and Related Substance Amendment Act (Steyn 2007) [43]	Forex fluctuation (depreciation of the local currency) (Walwyn 2018) [63]
		Unfavourable reimbursement practices (Ashigbie 2016) [49]

##### Micro level factors

Three studies cited education and awareness creation where prescribers and users were aware of the medicines and therapies under the new medicine pricing policy as facilitators of successful medicine pricing policies [43, 48, 56]. A key barrier to implementation of medicine pricing policies was the long distance travelled by individuals in order to access medicines [45, 51].

##### Meso level factors

Introduction of government subsidies and exemptions of generic medicines contributed to a decline in the prices of medicines at facility level in Mali [42]. Pooling resources and buying in bulk also reduced the prices of medicines in Ghana [67]. Shortage of trained personnel and resources to assess and scrutinize prices of medicines and lack of information about medicine prices by the pharmaceutical companies in Sudan [39], and limited access to medicines and frequent stockouts in multiple countries [40, 44, 51, 56], were the main barriers reported.

##### Macro level factors

The review showed that the use of national essential medicine lists by health facilities was a facilitator of the implementation of reimbursement schemes in Ghana [67]. Other facilitators were funding support from donor agencies [41, 49, 51], international policy interventions such as supporting domestic production of medicines [68] and existing national essential medicines list and medicine and related substance amendment Act [46, 67]. Challenges to implementation included lack of scrutiny on medicines pricing information by regulators with medicine prices of certain generics higher than their originators [39]. Additionally, unfavourable National Health Insurance Scheme reimbursement practices such as reimbursement delays and lack of price ‘mark-up’ standardization [67], and the lack of a coherent and well-functioning national medicine pricing policy, constrain efforts to regulate and ensure better prices for improved access [68].

#### Effectiveness of implementing medicine pricing policies

Medicine pricing policies sought to achieve two main aims: (a) control or reduce the prices of medicines and (b) improve access to essential medicines. Some studies reported separate effects on medicine prices [50, 53–55, 57, 59, 61, 62, 66] or improved access to essential medicines [45, 48, 58, 60]. However, as shown in Table 2, many studies reported on both effects [40–44, 46, 47, 49, 51, 56, 64, 65, 67].

##### Effect on prices of medicines

Overall, implementation of the different medicine pricing policies largely suggests a reduction in the prices of medicines. The results of the review showed that implementation of the tiered pricing (segmented pricing based on targeted markets), voluntary licensing (removing of regulatory barriers) and generic policy (promoting prescribing generic medicines) in seven different countries across Africa led to reductions in generic direct-acting antivirals (DAA) from US $1200 to between US $684 and US $750, i.e. the generic medicines were 40% cheaper than the originator prices [39]. However, some medicine pricing policies did not change much or appear to influence the prevailing medicine prices following implementation [50]. Although one of the goals of the Pharmacy and Poisons Act (2001) implemented in Sudan was to control prices of the medicines through regulating mark-ups along the supply chain, the evidence generated revealed that the policy did not appear to have an effect in the prevailing medicine prices [39].

##### Effects on improved access to medicines

Fifteen studies reported evidence of policy effect on improved availability and affordability to essential medicines [40–49, 51, 56, 60, 65, 69]. In Kenya, implementation of a revolving fund pharmacy model improved the availability of essential medicines from 30 to 40% to over 90% in 15 health facilities [45]. In South Africa, when generic reference pricing was implemented, the use of generic rosuvastatin increased from 24 to 63.9% in the subsequent year and to 76.4% a year later [47]. In their assessment of the use of artemisinin combination therapy for malaria across different households in Tanzania, the researchers found that artemisinin-based combination treatments increased availability within the retail sector from 31 to 49% and then to 61% [48].

## Discussion

This systematic review sought to identify medicines pricing policies implemented in SSA, how these were implemented, which contextual facilitators and barriers affected policy implementation and how effective were these policies. The review revealed 13 different medicine pricing policies reflecting four domains, targeted public subsidies, regulatory framework and direct price control, generic medicine policies and purchasing policies, were implemented across SSA between 2003 and 2020. The medicines pricing policies were implemented in less than half (22/46) of SSA countries. The main implementation approaches involved the use of regulatory frameworks and private distribution outlets and networks. The review showed key actors involved in policy implementation were government, wholesalers, manufacturers, retailers, professional bodies, community members and private and public health facilities. The use of evidence to inform policy implementation was not reported in any of the included studies. Key barriers to policy implementation identified included limited awareness about policies, frequent stock out, lack of capacity to regulate implementation and lack of price transparency in external reference pricing process, whereas key facilitators included existing national essential medicine policy environment, strong political will and support from development partners. Evidence on effectiveness of implemented policies on reducing prices and improving access to medicines was mixed. Reductions in prices were reported only in some studies. There was evidence that implementation of medicine pricing policy led to improved availability and affordability to essential medicines.

The categories of medicine pricing policies identified in this study are similar to what were previously reported, which also highlight predominant focus on regulatory measures or direct price control [70]. Although commonly reported in SSA, regulatory measure or direct control is considered highly contentious with no consensus in the literature. For example, opponents from the pharmaceutical sector advocate for a need for free and open market systems, arguing that government control undermines competition and innovation for developing new drugs and limits access in the market to address new medical conditions [71–73]. Weak systems for government direct price control may not lead to the required outcome. As revealed in Sudan [39] and the Philippines [74], regulation of medicine prices does not guarantee reduction in the prices of essential medicines and improved availability and affordability. It is therefore important to critically examine appropriateness of implementation approaches for achieving outcomes within specific contexts. Adequate capacity to monitor and evaluate policy implementation and understand contextual influences on the implementation is therefore critical.

The use of generic medicines as a strategy to reduce prices and ensure improved access was widely employed in SSA, and the effect on price and expenditure favours the use of quality-assured generic medicines [8]. A review of generic medicine pricing policies in Europe revealed that policies for implementing generic medicines used different implementation mechanisms such as reductions in reference prices and prescription status of medicines [75]. In our review, we found similar approaches for generic medicine policy and regulatory frameworks.

Although information on the role of actors was provided in the studies reviewed, the actors’ interests, agendas, relative powers and networking arrangements such as alliance building were missing. This information can be revealed through stakeholder analyses [76–79] and is often critical to form a comprehensive understanding of policy implementation [80–83]. This highlights one outstanding gap in the published knowledge on the implementation of medicine pricing policies, thus representing agenda for further research.

The review showed slightly more reported barriers than facilitators to implementation of medicine pricing policies. This may reflect researchers’ bias in revealing more constraints in their investigations [84, 85], though this may also reflect a greater number of contextual inhibitors to the implementation of medicines pricing policies in SSA contexts. The latter can be a particularly important contribution to the field of health policy analysis and transferability of theoretical and practical lessons learned to other health (and non-health) policies.

The ultimate goal of medicine pricing policies is to ensure low and affordable medicine prices as revealed in our review. This finding is in line with another study from Asia which found similar price reductions following implementation of generic medicine pricing policy in Indonesia [86]. The authors reported that following the implementation of the policy, the prices of lowest price generic and innovator brands fell from 40 to 2200% between 2004 and 2010. A review of pharmaceutical pricing policies in developing countries also revealed a similar outcome of reduced medicine pricing policies [70].

Some policies, however, did not have any effect on the prices of medicine [39, 54, 58]. For example, the introduction of a free medicines policy in Cameroon to provide free ART for people living with HIV appeared not to have achieved the goal of improving access to medicines. It was reported that the policy resulted in shortages in supplies, and as result, few patients were able to get the second-line treatment. This was attributed to the fact that the policy did not ensure that adequate systems and infrastructure were in place to address increased demand and avert resultant challenges impeding access to ARTs [58]. This is not new as previous studies revealed that the implementation of generic medicine pricing policy in Europe resulted in higher prices, but higher prices also stimulated competition between generic medicines leading to prices reduction [75].

Implementation of medicine pricing policies can be mediated by different contextual facilitators or barriers. Our review has highlighted that key contextual barriers comprised weak enforcement or regulatory mechanisms, the absence of essential medicines list and the role of foreign exchange currency fluctuations. On the other hand, facilitators included raising awareness about implementation, existence of subsidies, use of essential medicine lists, establishing a fixed profit margin or percentage for manufacturers and the pivotal roles of supportive donor agencies and international policies and interventions. A study in China also revealed contextual barriers such as lack of enforcement of pricing regulations and policies, with authors encouraging strong governance structures and legal frameworks to ensure enforcement [87]. The monitoring and enforcement of medicines pricing frameworks need to be supported by well-trained and skilled personnel, which is often lacking in different SSA countries [39, 53].

This study also reported on the effectiveness of the medicine pricing policies in SSA. Our review showed that some medicine pricing policies have the potential to improve access to essential medicines [40–49, 56, 60, 65, 69, 88], control or reduce the prices of essential medicines [50, 53, 54, 57, 59, 61, 62, 66, 89] or have dual impact of improved access and controlled price effects [40–44, 46, 47, 49, 56, 64, 65, 67, 88]. However, evidence on the impact of the medicine pricing policies should be carefully interpreted as most of the studies were from nonexperimental or controlled studies, largely cross-sectional studies, e.g. [40–44]. Although a diversity of study designs was included in the review, which were primarily nonexperimental in design, the findings still provide developing and potential evidence of impact following the implementation of the different medicine pricing policies.

The findings from this review contribute to the field of policy analysis. Specifically, the taxonomies of the categories of policies (i.e. targeted public subsidies, regulatory framework and direct price control, generic medicine policy and purchasing policies) and actor groups (e.g. government, community members) involved in implementation and the distinction of micro, meso and macro levels context, which mirrors other policy studies [8, 9, 70, 90, 91].

### Implications for policy and future research

This review suggests three implications for improving implementation of medicine pricing policies in SSA, which can also be applied to other health policies in LMICs more generally. First, four broad groups of policy options are available for reducing medicine pricing: targeted public subsidies, regulatory frameworks and direct price controls, generic medicine policies and purchasing policies. However, it is important to design and apply the country-specific implementation mechanisms to avoid a ‘one-size-fits-all’ approach. Second, different stakeholders from both the public and private sectors can play important roles in the design and implementation of medicine pricing policies. Inclusive policy processes which allow representation of multiple voices of policy actors are, therefore, imperative to ensure sustainability of policy implementation, pooling of resources and collective ownership and acceptance. This is particularly pertinent to medicines pricing, given that the private (for profit) sector plays a major role in pharmaceutical manufacturing and distribution, but it is also important to encourage participation of underrepresented not-for-profit groups such as civil society organizations, in health policy processes. Third, it is important to continuously monitor and evaluate the implementation approaches and emerging effects of these policies, something which our review observed was generally lacking. This can represent an opportunity for enhancing the culture of evidence-informed decision-making within government agencies, as well as closer partnerships between government agencies and research organizations.

We call for more research on medicine pricing policy implementation, covering three areas. First, more studies need to examine the role of evidence in the design and implementation of medicines pricing policies. The increased interest and attention on evidence-informed policy and planning decisions [90, 92–95] can sustain the momentum, and it is important to strengthen capacity within mainstream information systems to generate robust evidence rather than continuously rely on one-off and ‘external’ assessments [90, 93, 96]. Second, future research on the role of policy actors involved in the policy design process is critical for improving policy implementation, particularly covering actors’ interests, agendas, powers and resultant influences [80–83]. Third, it is critical to generate robust evidence on key contextual influences on policy implementation and understand how individual factors can facilitate or constrain implementation in different settings [97–100].

### Study limitations

We acknowledge the following limitations. First, the review focused on studies conducted in SSA, but we acknowledge variation in income status, socio-economic contexts and healthcare systems across countries. Different contexts inevitably affected how medicine pricing policies were implemented and their outcomes. As a result, we were guided by the generally limited contextual information included in the reviewed studies and resisted making too many assumptions and inferences based on our knowledge of the different countries. We also suggest that experimental studies could report more robust and less biased results, as compared to the reported studies in this review which were largely cross-sectional with limited follow-up. Second, we conducted comprehensive searches in a range of health science, global health and multidisciplinary databases to capture all SSA medicines pricing literature, and although we may have missed some potentially relevant studies by not including specific pharmaceutical databases such as International Pharmaceutical Abstracts, we believe that full articles of most of these abstracts would have been captured from at least one of the databases, and this omission is not expected to miss studies that could likely change the magnitude, direction or conclusions of this review. Third, given the different study designs employed, sample sizes and outcome measures, we faced a challenge to analytically compare the outcomes or effects of the policies on the prices and access to essential medicines. This notwithstanding, we feel our analysis provides a useful taxonomy of types of medicines pricing policies and highlights implementation approaches to inform future policy, practice and research.

## Conclusion

The implementation of medicine pricing policies in SSA focused on four policy options: targeted public subsidies, regulatory framework and direct price control, generic medicine policies and purchasing policies. Implementation of these policies in SSA shows some mixed evidence of improved availability and affordability to essential medicines, and it is important to understand country-specific experiences, diversity of policy actors and contextual barriers and facilitators to policy implementation. Our study suggests three policy implications for improving implementation of medicines pricing policies in SSA: avoiding ‘one-size-fits-all’ approach, engaging both private and public sector policy actors in policy implementation and continuously monitoring implementation and effects of policies. Future studies can usefully examine interests, influences, relative powers and coalition formation of policy actors during implementation of medicine pricing policies.

## Supplementary Information

**Additional file 1.** African Index Medicus (AIM).

**Additional file 2.** Screening flowchart.

**Additional file 3.** Quality assessment of included studies.

## Data Availability

All data generated or analysed during this study are included in this published article.

## Abbreviations

LMICs: Low- and middle-income countries
SSA: Sub-Saharan Africa
