# Supplementary material for: Implementation of medicines pricing policies in sub-Saharan Africa: systematic review
Source: Syst Rev. 2022 Dec 1;11:257. doi: 10.1186/s13643-022-02114-z (PMC9714131; doi:10.1186/s13643-022-02114-z)
Supplement: Supplementary file 1 — Additional file 1. African Index Medicus (AIM). [file 13643_2022_2114_MOESM1_ESM.docx]

# African Index Medicus (AIM)

<https://www.globalindexmedicus.net/>

Searched via Global Index Medicus interface: using Advanced search ti, ab,subject

Search Date: 25 May 2021

Filter = AIM; Filter + 2000 -2020. Two searches performed as cost concept was too large.

1. (tw:(drug* or prescription* or medicine* or pharmaceutical*)) **AND** (tw:(cost* OR costs OR price* OR pricing OR Afford* OR reimburs* OR generic* OR purchas* OR procur* OR expenditure* )) **AND** (tw:(policy OR policies OR guideline OR guidance OR law OR regulat* OR rule OR legislat* OR Control* OR strateg* OR framework* OR intervention* OR plan* OR program* OR tax OR taxes OR exemption*)) **AND** (tw:(implement* OR approach* OR Process* OR barrier* OR facilitator* OR challenge* OR motivator* OR factor* OR determinant* OR context* OR “scale up” OR “scaled up” OR “Scaling up” OR Adopt*)) = 90
2. (tw:((drug* or prescription* or medicine* or pharmaceutical*))) **AND** (tw:((subsid* OR tariff* OR incentive* OR containment OR transparency OR fee OR fees OR rebate* OR payment* OR spend* OR saving* OR Benchmark* OR cost-plus OR “essential medicine*” OR “essential drug”))) **AND** (tw:((policy OR policies OR guideline OR guidance OR law OR regulat* OR rule OR legislat* OR Control* OR strateg* OR framework* OR intervention* OR plan* OR program* OR tax OR taxes OR exemption*))) **AND** (tw:((implement* OR approach* OR Process* OR barrier* OR facilitator* OR challenge* OR motivator* OR factor* OR determinant* OR context* OR “scale up” OR “scaled up” OR “Scaling up” OR Adopt*))) = 181

## Embase (Ovid)<1996 to 2021 Week 20>

Search Date: 25 May 2021

--------------------------------------------------------------------------------

1 exp "Africa south of the Sahara"/ (227922)

2 (angola* or benin* or botswana* or "burkina faso" or burundi*).ti,ab,in,ad,kw. (26205)

3 ("cabo verde*" or "cape verde*" or cameroon* or "central africa*" or chad or cormoros or congo* or "ivory coast" or "cote d'ivoire" or djibouti).ti,ab,in,ad,kw. (45648)

4 (guinea* or eritrea* or eswatini* or swaziland* or ethiopia* or gabon* or gambia* or ghana* or guinea*).ti,ab,in,ad,kw. (108734)

5 (kenya* or lesotho* or liberia* or madagasca* or malawi* or mali or mauritania* or mauritius or mozambique*).ti,ab,in,ad,kw. (68324)

6 (namibia* or niger or nigeria* or rwanda*).ti,ab,in,ad,kw. (86856)

7 ("sao tome" or principe* or senegal* or seychelles or "sierra leone*" or somali* or "south africa*" or sudan*).ti,ab,in,ad,kw. (177476)

8 (tanzania* or togo* or uganda* or zambia* or zaire* or zimbabw*).ti,ab,in,ad,kw. (62485)

9 (africa* adj2 ("sub sahara*" or "south* sahara*")).ti,ab,in,ad,kw. (32543)

10 or/1-9 [sub-saharan africa] (522800)

11 "drug cost"/ (73602)

12 ((price? or pricing) adj5 (medicine? or drug? or prescription? or pharmaceutical*)).tw,kw. (8449)

13 ((cost or costs) adj5 (medicine? or drug? or prescription? or pharmaceutical*)).tw,kw. (34144)

14 (afford* adj5 (medicine? or drug? or prescription? or pharmaceutical*)).tw,kw. (2878)

15 (reimburs* adj5 (medicine? or drug? or prescription? or pharmaceutical*)).tw,kw. (4672)

16 (generic* adj5 (medicine? or drug? or prescription? or pharmaceutical*)).tw,kw. (8497)

17 generic drug/ (12702)

18 ((purchas* or procur* or expenditure*) adj5 (medicine? or drug? or prescription? or pharmaceutical*)).tw,kw. (7965)

19 ((subsid* or tariff* or incentive* or containment or transparency) adj5 (medicine? or drug? or prescription? or pharmaceutical*)).tw,kw. (3136)

20 ((fee or fees or rebate* or payment* or spend* or saving*) adj5 (medicine? or drug? or prescription? or pharmaceutical*)).tw,kw. (6766)

21 ((benchmark* or cost-plus) adj12 (medicine? or drug? or prescription? or pharmaceutical*)).tw,kw. (1204)

22 "low* price* generic*".tw,kw. (124)

23 (essential adj2 (drug* or medicine*)).tw,kw. (5465)

24 essential drug/ (1376)

25 (access* adj3 (medicine? or drug? or prescription? or pharmaceutical*)).tw,kw. (9867)

26 pharmacoeconomics/ (8364)

27 (pharma* adj2 economic*).tw,kw. (1228)

28 pharmacoeconomic*.tw,kw. (8938)

29 or/11-28 [drug pricing] (143775)

30 health care policy/ (176174)

31 exp drug control/ (61125)

32 ((drug* or medicine* or pharmaceutical* or prescription* or health*) adj7 (guideline* or guidance or policy or policies or law or regulat* or rule* or legislat* or control* or strateg* or framework*)).tw,kw. (774652)

33 (tax or taxes or exemption*).tw,kw. (17692)

34 ((drug* or medicine* or pharmaceutical* or prescription* or health*) adj7 (intervention* or plan* or program*)).tw,kw. (336046)

35 Government Regulation/ (22193)

36 or/30-35 [ policy concept] (1246409)

37 10 and 29 and 36 [SSA and policy] (2211)

38 exp program evaluation/ (27174)

39 (barrier* or facilitator* or challenge* or motivator*).tw,kw. (1198525)

40 (implement* or approach* or process*).tw,kw. (4815460)

41 (factor* or determinant* or context*).tw,kw. (4781918)

42 "scal* up".tw,kw. (26745)

43 scale-up/ (12389)

44 adopt*.tw,kw. (314371)

45 or/38-44 [implementation] (9354132)

46 10 and 29 and 36 and 45 (1377)

47 limit 46 to yr="2000 -Current" (1340)

## Global Health (Ovid)<1973 to 2021 Week 20>

Search Date: 25 May 2021

--------------------------------------------------------------------------------

1 exp "africa south of sahara"/ (186562)

2 (angola* or benin* or botswana* or "burkina faso" or burundi*).ti,ab,in,gl. (14740)

3 ("cabo verde*" or "cape verde*" or cameroon* or "central africa*" or chad or cormoros or congo* or "ivory coast" or "cote d'ivoire" or djibouti).ti,ab,in,gl. (26974)

4 (guinea* or eritrea* or eswatini* or swaziland* or ethiopia* or gabon* or gambia* or ghana* or guinea*).ti,ab,in,gl. (55448)

5 (kenya* or lesotho* or liberia* or madagasca* or malawi* or mali or mauritania* or mauritius or mozambique*).ti,ab,in,gl. (38754)

6 (namibia* or niger or nigeria* or rwanda*).ti,ab,in,gl. (59616)

7 ("sao tome" or principe* or senegal* or seychelles or "sierra leone*" or somali* or "south africa*" or sudan*).ti,ab,in,gl. (61361)

8 (tanzania* or togo* or uganda* or zambia* or zaire* or zimbabw*).ti,ab,in,gl. (38490)

9 (africa* adj2 ("sub sahara*" or "south* sahara*")).ti,ab,in,gl. (17508)

10 or/1-9 [sub-saharan africa] (260708)

11 (prescriptions/ or drugs/) and (costs/ or fees/) (695)

12 ((price? or pricing) adj5 (medicine? or drug? or prescription? or pharmaceutical*)).tw,id. (1045)

13 ((cost or costs) adj5 (medicine? or drug? or prescription? or pharmaceutical*)).tw,id. (4792)

14 (afford* adj5 (medicine? or drug? or prescription? or pharmaceutical*)).tw,id. (824)

15 (reimburs* adj5 (medicine? or drug? or prescription? or pharmaceutical*)).tw,id. (398)

16 (generic* adj5 (medicine? or drug? or prescription? or pharmaceutical*)).tw,id. (931)

17 generics/ (364)

18 ((purchas* or procur* or expenditure*) adj5 (medicine? or drug? or prescription? or pharmaceutical*)).tw,id. (1677)

19 ((subsid* or tariff* or incentive* or containment or transparency) adj5 (medicine? or drug? or prescription? or pharmaceutical*)).tw,id. (429)

20 ((fee or fees or rebate* or payment* or spend* or saving*) adj5 (medicine? or drug? or prescription? or pharmaceutical*)).tw,id. (812)

21 ((benchmark* or cost-plus) adj12 (medicine? or drug? or prescription? or pharmaceutical*)).tw,id. (76)

22 "low* price* generic*".tw,id. (51)

23 (essential adj2 (drug* or medicine*)).tw,id. (1634)

24 (access* adj3 (medicine? or drug? or prescription? or pharmaceutical*)).tw,id. (1966)

25 (pharma* adj2 economic*).tw,id. (141)

26 pharmacoeconomic*.tw,id. (374)

27 or/11-26 [drug pricing] (11818)

28 policy/ or health policy/ (38180)

29 legislation/ (11358)

30 regulations/ (13049)

31 ((drug* or medicine* or pharmaceutical* or prescription* or health*) adj7 (guideline* or guidance or policy or policies or law or regulat* or rule* or legislat* or control* or strateg* or framework*)).tw,id. (202358)

32 (tax or taxes or exemption*).tw,id. (4824)

33 ((drug* or medicine* or pharmaceutical* or prescription* or health*) adj7 (intervention* or plan* or program*)).tw,id. (354671)

34 or/28-33 [policy concept] (543006)

35 10 and 27 and 34 (1107)

36 program evaluation/ (659)

37 (barrier* or facilitator* or challenge* or motivator*).tw,id. (168576)

38 (implement* or approach* or process*).tw,id. (577865)

39 (factor* or determinant* or context*).tw,id. (835181)

40 "scal* up".tw,id. (8178)

41 adopt*.tw,id. (49436)

42 or/36-41 [implementation] (1363901)

43 10 and 27 and 34 and 42 (678)

44 limit 43 to yr="2000 -Current" (639)

## Ovid MEDLINE(R) ALL <1946 to May 24, 2021>

Search Date: 25 May 2021

--------------------------------------------------------------------------------

1 Drugs, Generic/ (5442)

2 exp "Africa South of the Sahara"/ (221405)

3 (angola* or benin* or botswana* or "burkina faso" or burundi*).ti,ab,in,kf. (20189)

4 ("cabo verde*" or "cape verde*" or cameroon* or "central africa*" or chad or cormoros or congo* or "ivory coast" or "cote d'ivoire" or djibouti).ti,ab,in,kf. (50720)

5 (guinea* or eritrea* or eswatini* or swaziland* or ethiopia* or gabon* or gambia* or ghana* or guinea*).ti,ab,in,kf. (169119)

6 (kenya* or lesotho* or liberia* or madagasca* or malawi* or mali or mauritania* or mauritius or mozambique*).ti,ab,in,kf. (62325)

7 (namibia* or niger or nigeria* or rwanda*).ti,ab,in,kf. (75810)

8 ("sao tome" or principe* or senegal* or seychelles or "sierra leone*" or somali* or "south africa*" or sudan*).ti,ab,in,kf. (149159)

9 (tanzania* or togo* or uganda* or zambia* or zaire* or zimbabw*).ti,ab,in,kf. (56911)

10 (africa* adj2 ("sub sahara*" or "south* sahara*")).ti,ab,in,kf. (27501)

11 or/2-10 [sub-saharan africa] (567162)

12 Drug Costs/ (16589)

13 ((price? or pricing) adj5 (medicine? or drug? or prescription? or pharmaceutical*)).tw,kw. (4947)

14 ((cost or costs) adj5 (medicine? or drug? or prescription? or pharmaceutical*)).tw,kw. (20270)

15 (afford* adj5 (medicine? or drug? or prescription? or pharmaceutical*)).tw,kw. (2142)

16 (reimburs* adj5 (medicine? or drug? or prescription? or pharmaceutical*)).tw,kw. (2267)

17 (generic* adj5 (medicine? or drug? or prescription? or pharmaceutical*)).tw,kw. (4814)

18 Drugs, Generic/ (5442)

19 exp fees, pharmaceutical/ (2462)

20 ((purchas* or procur* or expenditure*) adj5 (medicine? or drug? or prescription? or pharmaceutical*)).tw,kw. (5286)

21 ((subsid* or tariff* or incentive* or containment or transparency) adj5 (medicine? or drug? or prescription? or pharmaceutical*)).tw,kw. (2282)

22 ((fee or fees or rebate* or payment* or spend* or saving*) adj5 (medicine? or drug? or prescription? or pharmaceutical*)).tw,kw. (4351)

23 ((benchmark* or cost-plus) adj12 (medicine? or drug? or prescription? or pharmaceutical*)).tw,kw. (761)

24 "low* price* generic*".tw,kw. (86)

25 (essential adj2 (drug* or medicine*)).tw,kw. (3826)

26 Drugs, Essential/ (929)

27 (access* adj3 (medicine? or drug? or prescription? or pharmaceutical*)).tw,kw. (6689)

28 Economics, Pharmaceutical/ (2990)

29 (pharma* adj2 economic*).tw,kw. (893)

30 pharmacoeconomic*.tw,kw. (4110)

31 or/12-30 [drug pricing] (65258)

32 exp policy/ (162346)

33 Government Regulation/ (21532)

34 exp Legislation, Drug/ (33360)

35 ((drug* or medicine* or pharmaceutical* or prescription* or health*) adj7 (guideline* or guidance or policy or policies or law or regulat* or rule* or legislat* or control* or strateg* or framework*)).tw,kw. (593418)

36 (tax or taxes or exemption*).tw,kw. (18530)

37 ((drug* or medicine* or pharmaceutical* or prescription* or health*) adj7 (intervention* or plan* or program*)).tw,kw. (283851)

38 or/32-37 [policy concept -all] (1002267)

39 11 and 31 and 38 [SSA and policy ] (1336)

40 Health Plan Implementation/ (6408)

41 Program Evaluation/ (64911)

42 (barrier* or facilitator* or challenge* or motivator*).tw,kw. (1036117)

43 (implement* or approach* or process*).tw,kw. (4335406)

44 (factor* or determinant* or context*).tw,kw. (4148932)

45 "scal* up".tw,kw. (22149)

46 adopt*.tw,kw. (273188)

47 or/40-46 [implementation] (8374962)

48 11 and 31 and 38 and 47 (860)

49 limit 48 to yr="2000 -Current" (749)

## Web of Science Core Collection: Citation Indexes (Clarivate Analytics)

Search Date: 25 May 2021

Simultaneously searched the following:

- *Science Citation Index Expanded (SCI-EXPANDED) --1900-present*
- *Social Sciences Citation Index (SSCI) --1900-present*
- *Arts & Humanities Citation Index (A&HCI) --1975-present*
- *Conference Proceedings Citation Index- Science (CPCI-S) --1990-present*
- *Conference Proceedings Citation Index- Social Science & Humanities (CPCI-SSH) --1990-present*
- *Emerging Sources Citation Index (ESCI) --2015-present*

*Data last updated: 2021-05-25*

# 36 574 #34 AND #27 AND #23 AND #8 Indexes=SCI-EXPANDED, SSCI, A&HCI, CPCI-S, CPCI-SSH, ESCI Timespan=2000-2021

# 35 611 #34 AND #27 AND #23 AND #8

# 34 18,873,614 #33 OR #32 OR #31 OR #30 OR #29

# 33 884,966 TOPIC: (adopt*)

# 32 56,000 TOPIC: ("scal* up")

# 31 7,465,121 TOPIC: (factor* or determinant* or context*)

# 30 11,915,500 TOPIC: (implement* or approach* or process*)

# 29 2,160,951 TOPIC: (barrier* or facilitator* or challenge* or motivator*)

# 28 892 #27 AND #23 AND #8

# 27 969,995 #26 OR #25 OR #24

# 26 290,653 TOPIC: ((drug* or medicine* or pharmaceutical* or prescription* or health*) NEAR/7 (intervention* or plan* or program*) )

# 25 109,283 TOPIC: ((tax or taxes or exemption*) )

# 24 628,941 TOPIC: ((drug* or medicine* or pharmaceutical* or prescription* or health*) NEAR/7 (guideline* or guidance or policy or policies or law or regulat* or rule* or legislat* or control* or strateg* or framework*) )

# 23 34,696 #22 OR #21 OR #20 OR #19 OR #18 OR #17 OR #16 OR #15 OR #14 OR #13 OR #12 OR #11 OR #10 OR #9

# 22 5,234 TOPIC: (pharmacoeconomic*)

# 21 1,364 TOPIC: ((pharma* near/2 economic*) .)

# 20 4,791 TOPIC: ((access* near/3 (medicine? or drug? or prescription? or pharmaceutical*) ))

# 19 5,563 TOPIC: ((essential near/2 (drug* or medicine*) ))

# 18 83 TOPIC: ("low* price* generic*")

# 17 308 TOPIC: (((benchmark* or cost-plus) near/12 (medicine? or drug? or prescription? or pharmaceutical*) ))

# 16 2,154 TOPIC: (((fee or fees or rebate* or payment* or spend* or saving*) near/5 (medicine? or drug? or prescription? or pharmaceutical*) ).)

# 15 1,185 TS=(((subsid* or tariff* or incentive* or containment or transparency) near/5 (medicine? or drug? or prescription? or pharmaceutical*) ))

# 14 3,087 TOPIC: (((purchas* or procur* or expenditure*) near/5 (medicine? or drug? or prescription? or pharmaceutical*) ))

# 13 3,985 TOPIC: ((generic* near/5 (medicine? or drug? or prescription? or pharmaceutical*) ))

# 12 1,402 TOPIC: ((reimburs* near/5 (medicine? or drug? or prescription? or pharmaceutical*) ))

# 11 1,327 TOPIC: ((afford* near/5 (medicine? or drug? or prescription? or pharmaceutical*) ))

# 10 8,857 TOPIC: (((cost or costs) near/5 (medicine? or drug? or prescription? or pharmaceutical*) ))

# 9 3,114 TOPIC: (((price? or pricing) near/5 (medicine? or drug? or prescription? or pharmaceutical*) ))

# 8 1,094,912 #7 OR #6 OR #5 OR #4 OR #3 OR #2 OR #1

# 7 45,692 TOPIC: ((africa* near/2 ("sub sahara*" or "south* sahara*") )) OR ADDRESS: ((africa* near/2 ("sub sahara*" or "south* sahara*") ))

# 6 121,712 TOPIC: ((tanzania* or togo* or uganda* or zambia* or zaire* or zimbabw*) ) OR ADDRESS: ((tanzania* or togo* or uganda* or zambia* or zaire* or zimbabw*) )

# 5 523,885 TOPIC: (("sao tome" or principe* or senegal* or seychelles or "sierra leone*" or somali* or "south africa*" or sudan*) ) OR ADDRESS: (("sao tome" or principe* or senegal* or seychelles or "sierra leone*" or somali* or "south africa*" or sudan*) )

# 4 146,309 TOPIC: ((kenya* or lesotho* or liberia* or madagasca* or malawi* or mali or mauritania* or mauritius or mozambique*) ) OR ADDRESS: ((kenya* or lesotho* or liberia* or madagasca* or malawi* or mali or mauritania* or mauritius or mozambique*) )

# 3 276,150 TOPIC: ((guinea* or eritrea* or eswatini* or swaziland* or ethiopia* or gabon* or gambia* or ghana* or guinea*) ) OR ADDRESS: ((guinea* or eritrea* or eswatini* or swaziland* or ethiopia* or gabon* or gambia* or ghana* or guinea*) )

# 2 84,624 TOPIC: (("cabo verde*" or "cape verde*" or cameroon* or "central africa*" or chad or cormoros or congo* or "ivory coast" or "cote d'ivoire" or djibouti) ) OR ADDRESS: (("cabo verde*" or "cape verde*" or cameroon* or "central africa*" or chad or cormoros or congo* or "ivory coast" or "cote d'ivoire" or djibouti) )

# 1 49,487 TOPIC: ((angola* or benin* or botswana* or "burkina faso" or burundi*) ) OR ADDRESS: ((angola* or benin* or botswana* or "burkina faso" or burundi*) )

## Scopus (Elsevier B.V) 1823 to present

<https://www.scopus.com/>

Search Date: 25 May 2021

31 ( ( ( TITLE-ABS-KEY ( angola* OR benin* OR botswana* OR "burkina faso" OR burundi* ) ) OR ( AFFIL ( angola* OR benin* OR botswana* OR "burkina faso" OR burundi* ) ) ) OR ( ( TITLE-ABS-KEY ( "cabo verde*" OR "cape verde*" OR cameroon* OR "central africa*" OR chad OR cormoros OR congo* OR "ivory coast" OR "cote d'ivoire" OR djibouti ) ) OR ( AFFIL ( "cabo verde*" OR "cape verde*" OR cameroon* OR "central africa*" OR chad OR cormoros OR congo* OR "ivory coast" OR "cote d'ivoire" OR djibouti ) ) ) OR ( ( TITLE-ABS-KEY ( eritrea* OR eswatini* OR swaziland* OR ethiopia* OR gabon* OR gambia* OR ghana* OR guinea* ) ) OR ( AFFIL ( eritrea* OR eswatini* OR swaziland* OR ethiopia* OR gabon* OR gambia* OR ghana* OR guinea* ) ) ) OR ( ( TITLE-ABS-KEY ( kenya* OR lesotho* OR liberia* OR madagasca* OR malawi* OR mali OR mauritania* OR mauritius OR mozambique* ) ) OR ( AFFIL ( kenya* OR lesotho* OR liberia* OR madagasca* OR malawi* OR mali OR mauritania* OR mauritius OR mozambique* ) ) ) OR ( ( TITLE-ABS-KEY ( "sao tome" OR principe* OR senegal* OR seychelles OR "sierra leone*" OR somali* OR "south africa*" OR sudan* ) ) OR ( AFFIL ( "sao tome" OR principe* OR senegal* OR seychelles OR "sierra leone*" OR somali* OR "south africa*" OR sudan* ) ) ) OR ( ( TITLE-ABS-KEY ( tanzania* OR togo* OR uganda* OR zambia* OR zaire* OR zimbabw* ) ) OR ( AFFIL ( tanzania* OR togo* OR uganda* OR zambia* OR zaire* OR zimbabw* ) ) ) OR ( ( TITLE-ABS-KEY ( africa* W/2 ( "sub sahara*" OR "south* sahara*" ) ) ) OR ( AFFIL ( africa* W/2 ( "sub sahara*" OR "south* sahara*" ) ) ) ) ) AND ( ( TITLE-ABS ( ( ( cost OR price OR pricing OR afford* OR reimburse* OR generic ) W/5 ( medicine OR drug OR prescription OR pharmaceutical ) ) ) ) OR ( TITLE-ABS-KEY ( ( ( purchas* OR procur* OR expenditure* OR subsid* OR tariff* OR incentive OR containment OR transparency ) W/5 ( medicine OR drug OR prescription OR pharmaceutical ) ) ) ) OR ( TITLE-ABS-KEY ( ( ( fee OR rebate OR payment OR spend* OR saving ) W/5 ( medicine OR drug OR prescription OR pharmaceutical ) ) ) ) OR ( TITLE-ABS-KEY ( ( ( benchmark* OR cost-plus ) W/12 ( medicine OR drug OR prescription OR pharmaceutical ) ) ) ) OR ( TITLE-ABS-KEY ( "low* price* generic*" ) ) OR ( TITLE-ABS-KEY ( essential W/2 ( drug OR medicine ) ) ) OR ( TITLE-ABS-KEY ( access* W/3 ( drug OR medicine OR prescription OR pharmaceutical ) ) ) OR ( TITLE-ABS-KEY ( pharma* W/2 economic* ) ) OR ( TITLE-ABS-KEY ( pharmacoeconomic* ) ) ) AND ( ( TITLE-ABS-KEY ( ( drug* OR medicine* OR pharmaceutical* OR prescription* OR health* ) W/7 ( guideline* OR guidance OR policy OR policies OR law OR regulat* OR rule* OR legislat* OR control* OR strateg* OR framework* ) ) ) OR ( TITLE-ABS-KEY ( ( drug* OR medicine* OR pharmaceutical* OR prescription* OR health* ) W/7 ( intervention* OR plan* OR program* ) ) ) OR ( TITLE-ABS-KEY ( tax OR taxes OR exemption* ) ) ) AND ( ( TITLE-ABS-KEY ( barrier* OR facilitator* OR challenge* OR motivator* ) ) OR ( TITLE-ABS-KEY ( implement* OR approach* OR process* ) ) OR ( TITLE-ABS-KEY ( factor* OR determinant* OR context* ) ) OR ( TITLE-ABS-KEY ( "scal* up" OR adopt* ) ) ) AND PUBYEAR > 1999 ... 1,129

29 ( ( ( TITLE-ABS-KEY ( angola* OR benin* OR botswana* OR "burkina faso" OR burundi* ) ) OR ( AFFIL ( angola* OR benin* OR botswana* OR "burkina faso" OR burundi* ) ) ) OR ( ( TITLE-ABS-KEY ( "cabo verde*" OR "cape verde*" OR cameroon* OR "central africa*" OR chad OR cormoros OR congo* OR "ivory coast" OR "cote d'ivoire" OR djibouti ) ) OR ( AFFIL ( "cabo verde*" OR "cape verde*" OR cameroon* OR "central africa*" OR chad OR cormoros OR congo* OR "ivory coast" OR "cote d'ivoire" OR djibouti ) ) ) OR ( ( TITLE-ABS-KEY ( eritrea* OR eswatini* OR swaziland* OR ethiopia* OR gabon* OR gambia* OR ghana* OR guinea* ) ) OR ( AFFIL ( eritrea* OR eswatini* OR swaziland* OR ethiopia* OR gabon* OR gambia* OR ghana* OR guinea* ) ) ) OR ( ( TITLE-ABS-KEY ( kenya* OR lesotho* OR liberia* OR madagasca* OR malawi* OR mali OR mauritania* OR mauritius OR mozambique* ) ) OR ( AFFIL ( kenya* OR lesotho* OR liberia* OR madagasca* OR malawi* OR mali OR mauritania* OR mauritius OR mozambique* ) ) ) OR ( ( TITLE-ABS-KEY ( "sao tome" OR principe* OR senegal* OR seychelles OR "sierra leone*" OR somali* OR "south africa*" OR sudan* ) ) OR ( AFFIL ( "sao tome" OR principe* OR senegal* OR seychelles OR "sierra leone*" OR somali* OR "south africa*" OR sudan* ) ) ) OR ( ( TITLE-ABS-KEY ( tanzania* OR togo* OR uganda* OR zambia* OR zaire* OR zimbabw* ) ) OR ( AFFIL ( tanzania* OR togo* OR uganda* OR zambia* OR zaire* OR zimbabw* ) ) ) OR ( ( TITLE-ABS-KEY ( africa* W/2 ( "sub sahara*" OR "south* sahara*" ) ) ) OR ( AFFIL ( africa* W/2 ( "sub sahara*" OR "south* sahara*" ) ) ) ) ) AND ( ( TITLE-ABS ( ( ( cost OR price OR pricing OR afford* OR reimburse* OR generic ) W/5 ( medicine OR drug OR prescription OR pharmaceutical ) ) ) ) OR ( TITLE-ABS-KEY ( ( ( purchas* OR procur* OR expenditure* OR subsid* OR tariff* OR incentive OR containment OR transparency ) W/5 ( medicine OR drug OR prescription OR pharmaceutical ) ) ) ) OR ( TITLE-ABS-KEY ( ( ( fee OR rebate OR payment OR spend* OR saving ) W/5 ( medicine OR drug OR prescription OR pharmaceutical ) ) ) ) OR ( TITLE-ABS-KEY ( ( ( benchmark* OR cost-plus ) W/12 ( medicine OR drug OR prescription OR pharmaceutical ) ) ) ) OR ( TITLE-ABS-KEY ( "low* price* generic*" ) ) OR ( TITLE-ABS-KEY ( essential W/2 ( drug OR medicine ) ) ) OR ( TITLE-ABS-KEY ( access* W/3 ( drug OR medicine OR prescription OR pharmaceutical ) ) ) OR ( TITLE-ABS-KEY ( pharma* W/2 economic* ) ) OR ( TITLE-ABS-KEY ( pharmacoeconomic* ) ) ) AND ( ( TITLE-ABS-KEY ( ( drug* OR medicine* OR pharmaceutical* OR prescription* OR health* ) W/7 ( guideline* OR guidance OR policy OR policies OR law OR regulat* OR rule* OR legislat* OR control* OR strateg* OR framework* ) ) ) OR ( TITLE-ABS-KEY ( ( drug* OR medicine* OR pharmaceutical* OR prescription* OR health* ) W/7 ( intervention* OR plan* OR program* ) ) ) OR ( TITLE-ABS-KEY ( tax OR taxes OR exemption* ) ) ) AND ( ( TITLE-ABS-KEY ( barrier* OR facilitator* OR challenge* OR motivator* ) ) OR ( TITLE-ABS-KEY ( implement* OR approach* OR process* ) ) OR ( TITLE-ABS-KEY ( factor* OR determinant* OR context* ) ) OR ( TITLE-ABS-KEY ( "scal* up" OR adopt* ) ) ) ... 1,271

28 ( TITLE-ABS-KEY ( barrier* OR facilitator* OR challenge* OR motivator* ) ) OR ( TITLE-ABS-KEY ( implement* OR approach* OR process* ) ) OR ( TITLE-ABS-KEY ( factor* OR determinant* OR context* ) ) OR ( TITLE-ABS-KEY ( "scal* up" OR adopt* ) ) 28,398,928

27 TITLE-ABS-KEY ( "scal* up" OR adopt* ) 1,380,543

26 TITLE-ABS-KEY ( factor* OR determinant* OR context* ) 11,625,313

25 TITLE-ABS-KEY ( implement* OR approach* OR process* ) 18,032,336

24 TITLE-ABS-KEY ( barrier* OR facilitator* OR challenge* OR motivator* ) 2,814,994

23 ( TITLE-ABS-KEY ( ( drug* OR medicine* OR pharmaceutical* OR prescription* OR health* ) W/7 ( guideline* OR guidance OR policy OR policies OR law OR regulat* OR rule* OR legislat* OR control* OR strateg* OR framework* ) ) ) OR ( TITLE-ABS-KEY ( ( drug* OR medicine* OR pharmaceutical* OR prescription* OR health* ) W/7 ( intervention* OR plan* OR program* ) ) ) OR ( TITLE-ABS-KEY ( tax OR taxes OR exemption* ) ) 1,690,296

22 TITLE-ABS-KEY ( tax OR taxes OR exemption* ) 124,296

21 TITLE-ABS-KEY ( ( drug* OR medicine* OR pharmaceutical* OR prescription* OR health* ) W/7 ( intervention* OR plan* OR program* ) ) 647,313

20 TITLE-ABS-KEY ( ( drug* OR medicine* OR pharmaceutical* OR prescription* OR health* ) W/7 ( guideline* OR guidance OR policy OR policies OR law OR regulat* OR rule* OR legislat* OR control* OR strateg* OR framework* ) ) 1,065,268

19 ( TITLE-ABS ( ( ( cost OR price OR pricing OR afford* OR reimburse* OR generic ) W/5 ( medicine OR drug OR prescription OR pharmaceutical ) ) ) ) OR ( TITLE-ABS-KEY ( ( ( purchas* OR procur* OR expenditure* OR subsid* OR tariff* OR incentive OR containment OR transparency ) W/5 ( medicine OR drug OR prescription OR pharmaceutical ) ) ) ) OR ( TITLE-ABS-KEY ( ( ( fee OR rebate OR payment OR spend* OR saving ) W/5 ( medicine OR drug OR prescription OR pharmaceutical ) ) ) ) OR ( TITLE-ABS-KEY ( ( ( benchmark* OR cost-plus ) W/12 ( medicine OR drug OR prescription OR pharmaceutical ) ) ) ) OR ( TITLE-ABS-KEY ( "low* price* generic*" ) ) OR ( TITLE-ABS-KEY ( essential W/2 ( drug OR medicine ) ) ) OR ( TITLE-ABS-KEY ( access* W/3 ( drug OR medicine OR prescription OR pharmaceutical ) ) ) OR ( TITLE-ABS-KEY ( pharma* W/2 economic* ) ) OR ( TITLE-ABS-KEY ( pharmacoeconomic* ) ) 84,481

18 TITLE-ABS-KEY ( pharmacoeconomic* ) 9,282

17 TITLE-ABS-KEY ( pharma* W/2 economic* ) 4,224

16 TITLE-ABS-KEY ( access* W/3 ( drug OR medicine OR prescription OR pharmaceutical ) ) 11,284

15 TITLE-ABS-KEY ( essential W/2 ( drug OR medicine ) ) 8,534

14 TITLE-ABS-KEY ( "low* price* generic*" ) 111

13 TITLE-ABS-KEY ( ( ( benchmark* OR cost-plus ) W/12 ( medicine OR drug OR prescription OR pharmaceutical ) ) ) 1,180

12 TITLE-ABS-KEY ( ( ( fee OR rebate OR payment OR spend* OR saving ) W/5 ( medicine OR drug OR prescription OR pharmaceutical ) ) ) 8,772

11 TITLE-ABS-KEY ( ( ( purchas* OR procur* OR expenditure* OR subsid* OR tariff* OR incentive OR containment OR transparency ) W/5 ( medicine OR drug OR prescription OR pharmaceutical ) ) ) 11,397

10 TITLE-ABS ( ( ( cost OR price OR pricing OR afford* OR reimburse* OR generic ) W/5 ( medicine OR drug OR prescription OR pharmaceutical ) ) ) 46,666

9 ( ( TITLE-ABS-KEY ( angola* OR benin* OR botswana* OR "burkina faso" OR burundi* ) ) OR ( AFFIL ( angola* OR benin* OR botswana* OR "burkina faso" OR burundi* ) ) ) OR ( ( TITLE-ABS-KEY ( "cabo verde*" OR "cape verde*" OR cameroon* OR "central africa*" OR chad OR cormoros OR congo* OR "ivory coast" OR "cote d'ivoire" OR djibouti ) ) OR ( AFFIL ( "cabo verde*" OR "cape verde*" OR cameroon* OR "central africa*" OR chad OR cormoros OR congo* OR "ivory coast" OR "cote d'ivoire" OR djibouti ) ) ) OR ( ( TITLE-ABS-KEY ( eritrea* OR eswatini* OR swaziland* OR ethiopia* OR gabon* OR gambia* OR ghana* OR guinea* ) ) OR ( AFFIL ( eritrea* OR eswatini* OR swaziland* OR ethiopia* OR gabon* OR gambia* OR ghana* OR guinea* ) ) ) OR ( ( TITLE-ABS-KEY ( kenya* OR lesotho* OR liberia* OR madagasca* OR malawi* OR mali OR mauritania* OR mauritius OR mozambique* ) ) OR ( AFFIL ( kenya* OR lesotho* OR liberia* OR madagasca* OR malawi* OR mali OR mauritania* OR mauritius OR mozambique* ) ) ) OR ( ( TITLE-ABS-KEY ( "sao tome" OR principe* OR senegal* OR seychelles OR "sierra leone*" OR somali* OR "south africa*" OR sudan* ) ) OR ( AFFIL ( "sao tome" OR principe* OR senegal* OR seychelles OR "sierra leone*" OR somali* OR "south africa*" OR sudan* ) ) ) OR ( ( TITLE-ABS-KEY ( tanzania* OR togo* OR uganda* OR zambia* OR zaire* OR zimbabw* ) ) OR ( AFFIL ( tanzania* OR togo* OR uganda* OR zambia* OR zaire* OR zimbabw* ) ) ) OR ( ( TITLE-ABS-KEY ( africa* W/2 ( "sub sahara*" OR "south* sahara*" ) ) ) OR ( AFFIL ( africa* W/2 ( "sub sahara*" OR "south* sahara*" ) ) ) ) 1,298,478

8 ( TITLE-ABS-KEY ( africa* W/2 ( "sub sahara*" OR "south* sahara*" ) ) ) OR ( AFFIL ( africa* W/2 ( "sub sahara*" OR "south* sahara*" ) ) ) 71,763

7 ( TITLE-ABS-KEY ( tanzania* OR togo* OR uganda* OR zambia* OR zaire* OR zimbabw* ) ) OR ( AFFIL ( tanzania* OR togo* OR uganda* OR zambia* OR zaire* OR zimbabw* ) ) 139,261

6 ( TITLE-ABS-KEY ( "sao tome" OR principe* OR senegal* OR seychelles OR "sierra leone*" OR somali* OR "south africa*" OR sudan* ) ) OR ( AFFIL ( "sao tome" OR principe* OR senegal* OR seychelles OR "sierra leone*" OR somali* OR "south africa*" OR sudan* ) ) 594,697

5 ( TITLE-ABS-KEY ( kenya* OR lesotho* OR liberia* OR madagasca* OR malawi* OR mali OR mauritania* OR mauritius OR mozambique* ) ) OR ( AFFIL ( kenya* OR lesotho* OR liberia* OR madagasca* OR malawi* OR mali OR mauritania* OR mauritius OR mozambique* ) ) 163,089

4 ( TITLE-ABS-KEY ( eritrea* OR eswatini* OR swaziland* OR ethiopia* OR gabon* OR gambia* OR ghana* OR guinea* ) ) OR ( AFFIL ( eritrea* OR eswatini* OR swaziland* OR ethiopia* OR gabon* OR gambia* OR ghana* OR guinea* ) ) 351,147

2 ( TITLE-ABS-KEY ( "cabo verde*" OR "cape verde*" OR cameroon* OR "central africa*" OR chad OR cormoros OR congo* OR "ivory coast" OR "cote d'ivoire" OR djibouti ) ) OR ( AFFIL ( "cabo verde*" OR "cape verde*" OR cameroon* OR "central africa*" OR chad OR cormoros OR congo* OR "ivory coast" OR "cote d'ivoire" OR djibouti ) ) 109,449

1 ( TITLE-ABS-KEY ( angola* OR benin* OR botswana* OR "burkina faso" OR burundi* ) ) OR ( AFFIL ( angola* OR benin* OR botswana* OR "burkina faso" OR burundi* ) ) 62,238

# Alternative databases for grey literature

## WHO IRIS, Institutional Repository for Information Sharing

<https://apps.who.int/iris/>

Search Date: 28 May 2021 – no new titles since original search in 24 April 2020

Search interface: Regional Office for Africa Community: Advanced search

(drug OR drugs OR medicine OR medicines OR prescription OR prescriptions OR pharmaceutical OR pharmaceuticals) AND

Filter subject: cost* = 12 with date limit 2000: 5 (already in database)

Filter subject: reimburse = 1 with date limit 2000: 1 (already in database)

Filter subject: expenditure = 3 with date limit 2000: 3 (already in database)

Filter subject: access= 22 with date limit 2000: 21 (already in database)

Filter subject: price | Pricing | containment 0/2 (not drugs) |afford* |transparency| purchase )/6 – too old) | procure*| tariff* | incentive*| payment| incentive | subsidy|spend*| fee |rebate|benchmark* all gave 0 hits

Erudit (Observatoire des sciences et des technologies) all available dates <https://www.erudit.org/en/search/avancee/>

Search Date: 25 May 2021

[35 deduplicated by selecting for download in database]

Used English interface

**4** results found for the query:

(All fields : (angola* or benin* or botswana* or "burkina faso" or burundi* or "cabo verde*" or "cape verde*" or cameroon* or "central africa*" or chad or cormoros or congo* or "ivory coast" or "cote d'ivoire" or Djibouti or guinea* or eritrea* or eswatini* or swaziland* or ethiopia* or gabon* or gambia* or ghana* or guinea* or kenya*)) AND (Title, abstract, keywords : (drug* or prescription* or medicine* or pharmaceutical*) ) AND (Title, abstract, keywords : (cost* OR costs OR price* OR pricing OR Afford* OR reimburs* OR generic* OR purchas* OR procur* OR expenditure*)) AND (Published between 2000 and 2021) AND (Collections : ['Érudit', 'UNB'])

2 results found for the query:

(All fields : (lesotho* or liberia* or madagasca* or malawi* or mali or mauritania* or mauritius or mozambique* or namibia* or niger or nigeria* or Rwanda* )) AND (Title, abstract, keywords : (drug* or prescription* or medicine* or pharmaceutical*) ) AND (Title, abstract, keywords : (cost* OR costs OR price* OR pricing OR Afford* OR reimburs* OR generic* OR purchas* OR procur* OR expenditure*)) AND (Published between 2000 and 2021) AND (Collections : ['Érudit', 'UNB'])

0 results found for the query:

(All fields : (“sao tome" or principe* or senegal* or seychelles or "sierra leone*" or somali* or "south africa*" or sudan* or tanzania* or togo* or uganda* or zambia* or zaire* or zimbabw* or Africa* or sahara* or sub-saharan)) AND (Title, abstract, keywords : (drug* or prescription* or medicine* or pharmaceutical*) ) AND (Title, abstract, keywords : (cost* OR costs OR price* OR pricing OR Afford* OR reimburs* OR generic* OR purchas* OR procur* OR expenditure*)) AND (Published between 2000 and 2021) AND (Collections : ['Érudit', 'UNB'])

26 results found for the query:

(All fields : angola* or benin* or botswana* or "burkina faso" or burundi* or "cabo verde*" or "cape verde*" or cameroon* or "central africa*" or chad or cormoros or congo* or "ivory coast" or "cote d'ivoire" or Djibouti or guinea* or eritrea* or eswatini* or swaziland* or ethiopia* or gabon* or gambia* or ghana* or guinea* or kenya*) AND (Title, abstract, keywords : drug* or prescription* or medicine* or pharmaceutical*) AND (Title, abstract, keywords : subsid* OR tariff* OR incentive* OR containment OR transparency OR fee OR fees OR rebate* OR payment* OR spend* OR saving* OR Benchmark* OR cost-plus OR “essential medicine” OR “essential drug”) AND (Published between 2000 and 2021) AND (Collections : ['Érudit', 'UNB'])

14 results found for the query:

(All fields : lesotho* or liberia* or madagasca* or malawi* or mali or mauritania* or mauritius or mozambique* or namibia* or niger or nigeria* or Rwanda* ) AND (Title, abstract, keywords : drug* or prescription* or medicine* or pharmaceutical*) AND (Title, abstract, keywords : subsid* OR tariff* OR incentive* OR containment OR transparency OR fee OR fees OR rebate* OR payment* OR spend* OR saving* OR Benchmark* OR cost-plus OR “essential medicine” OR “essential drug”) AND (Published between 2000 and 2021) AND (Collections : ['Érudit', 'UNB'])0 result found by the query:

0 result found by the query:

(All fields : “sao tome" or principe* or senegal* or seychelles or "sierra leone*" or somali* or "south africa*" or sudan* or tanzania* or togo* or uganda* or zambia* or zaire* or zimbabw* or Africa* or sahara* or sub-saharan) AND (Title, abstract, keywords : drug* or prescription* or medicine* or pharmaceutical*) AND (Title, abstract, keywords : subsid* OR tariff* OR incentive* OR containment OR transparency OR fee OR fees OR rebate* OR payment* OR spend* OR saving* OR Benchmark* OR cost-plus OR “essential medicine” OR “essential drug”) AND (Published between 2000 and 2021) AND (Collections : ['Érudit', 'UNB'])

## Cairn International Database (Cairn Info) all available dates <https://www.cairn-int.info/>

Search Date: 25 May 2021

Used English Interface: Did not select articles that were obviously not relevant [non SSA] by title- although many will be irrelevant. Download 47 unique / 138 total in 3 searches [note: selection recognised preselected articles]. In update only selected new content.

47 from original search and 3 in update search = 50 /XXX

((drug* OR prescription* OR medicine* OR pharmaceutical*) w/5 (cost OR costs OR price OR pricing OR afford OR affordable OR reimburse OR reimbursement OR subsidy OR subsidies)) AND (policy OR policies OR strategy OR framework OR guideline OR guidance OR legislation OR regulation* OR tax OR taxes OR exemption*) = 86

((drug* or prescription* or medicine* or pharmaceutical*) w/5 (generic* OR purchase or purchasing OR procure or procurement OR expenditure* OR tariff OR tariffs)) AND (policy or policies or strategy or framework or guideline or guidance or legislation or regulation* or tax or taxes or exemptions) = 46

((drug* or prescription* or medicine* or pharmaceutical*) w/5 (incentive*or containment or transparency or fee or fees or spend* or saving*)) AND (policy or policies or strategy or framework* or guideline* or guidance or legislat* or regulat* or tax or taxes or exemption*) = 23

## World Bank Open Knowledge Repository

[**https://openknowledge.worldbank.org/**](https://openknowledge.worldbank.org/)

**Date searched 29 June 2021**

The 4 searches were repeated on the World Bank repository and the results merged to do a title and/or abstract screen. Duplicates were removed, then the remaining records were compared against the initial search results from June 2020 before the titles and were screened for inclusion into the first phase of screening. It should be noted that there appeared to be a change in the World Bank indexing tags. In June 2020 we used the Sub-Saharan Africa filter and retrieved 113 records in Search 1, in June 2021 the same search retrieved 14 records. Changing the filter to Africa retrieved 161 results. A pragmatic decision was made to change the filter to ‘Africa’ as several individual SSA country records (Ghana, Tanzania etc) were not retrieved when using the SSA filter. In the update, we also restricted the date from 2010 as older records would have been found in the initial search in June 2020.

| **Search string** | **June 2020**  **Searcher ACC (1to 2/6/20)**  **Filter: focus – sub Saharan Africa + years 2000 onwards + En +** | **June 2021(modified)**  **Searcher NK (29/6/21)**  **Filter: world bank region – Africa + years 2010 onwards + En** |
| --- | --- | --- |
| **Search 1**: (drug OR prescription OR medicine OR pharmaceutical) AND (cost OR pricing OR afford OR reimburse) | 2010-2019 (results 1-63) & 2000-2009 (results 64-113)= **113**  Repeated in June 2021(NK)  2020-2021 (results 1) 2010-2019 (results 9) & 2000-2009 (results 4)= 14 | 2010-2019 (results 87) & 2020-2021 (results 2) = **89** |
| **Search 2** (drug OR prescription OR medicine OR pharmaceutical) AND (purchase OR procurement OR Generic OR expenditure) | 2010-2019 (results 74) & 2000-2009 (results 75) = **149**  Repeated in June 2021  2020-2021 (results 0) 2010-2019 (results 12) & 2000-2009 (results 0)= 12 | 2010-2019 (results 101), 2020-2021 (results 0) = **101** |
| **Search 3:** (drug OR prescription OR medicine OR pharmaceutical) AND (purchase OR procurement OR Generic OR expenditure) | 2010-2019 (results 71) & 2000-2009 (results 56) = **127**  Repeated in June 2021  2020-2021 (results 0) 2010-2019 (results 8) & 2000-2009 (results 0)=  **8** | 2010-2019 (results 96) Africa, 2020-2021 ( results 1) **= 97** |
| **Search 4:** (drug OR prescription OR medicine OR pharmaceutical) AND (rebate or affordability) | 2010-2019 (Results 8) = **8**  Repeated in June 2021  2020-2021 (results 0) 2010-2019 (results 0) & 2000-2009 (results 0) = 0 | 2010-2019 (results 7), 2020-2021 (results 0) = **7** |
|  | **Total results = 397**  203 duplicates removed from all 4 searches  **Total to screen = 194**  **Included = 4**  **Maybes = 1**  Background & excluded = 3  Excluded = 186 | **Total results = 294**  151 duplicates removed from all 4 searches (n =143)with 99 records duplicated against 2020 search  **Total to screen = 44**  **Included = 0**  **Maybes = 0**  Background & excluded = 0  Excluded = 44 |

To calculate world bank total: 194 +44 = 238 records screened

397 + 294 = 691 retrieved

9 +0 (inc for screening/maybe or bkd)
