# Supplementary material for: Implementation of medicines pricing policies in sub-Saharan Africa: systematic review
Source: Syst Rev. 2022 Dec 1;11:257. doi: 10.1186/s13643-022-02114-z (PMC9714131; doi:10.1186/s13643-022-02114-z)
Supplement: Supplementary file 2 — Additional file 2. Screening flowchart. [file 13643_2022_2114_MOESM2_ESM.docx]

**A**dditional file 2: Screening flowchart

**Step 1: Does the study focus on**

**Drug/Medicines pricing?**

- Drug/Medicine pricing policy?
- Reference pricing?
- Pharmaceuticals / drugs / prescriptions cost / purchase?
- Affordability / fees / rebate / tariffs / incentives / payment?
- Benchmarking /expenditure / subsidy /procurement?

NO (other policies, not drug/medicine pricing)

Wrong topic - **EXCLUDE**

Label: not medicines pricing policy

YES

NO (agenda-setting or policy development)

**Step 2: Does study focus on Policy**

**Implementation?**

- Policy / strategy / plan / Guideline
- Implementation approach / Process?
- Facilitators / Barriers / Factors / Determinants / context?

Wrong topic - **EXCLUDE**

Label: not implementation

**SSA countries**

| **Angola**  **Benin**  **Botswana**  **Burkina Faso**  **Burundi**  **Cameroon**  **Cape Verde**  **Central African Republic**  **Chad**  **Comoros**  **Congo**  **Cote d'Ivoire**  **Djibouti**  **Equatorial Guinea**  **Ethiopia**  **Gabon**  **The Gambia**  **Ghana**  **Guinea**  **Guinea-Bissau**  **Kenya**  **Lesotho**  **Liberia** | **Madagascar**  **Malawi**  **Mali**  **Mauritania**  **Mauritius**  **Mozambique**  **Namibia**  **Niger**  **Nigeria**  **Rwanda**  **Sao Tome and Principe**  **Senegal**  **Seychelles**  **Sierra Leone**  **Somalia**  **South Africa**  **Sudan**  **Swaziland**  **Tanzania**  **Togo**  **Uganda**  **Zaire**  **Zambia**  **Zimbabwe** |
| --- | --- |

YES

**Step 4: Does the study focus on a**

**Country in sub-Saharan Africa?**

- See list of countries in SSA

Wrong setting

**EXCLUDE**

Label: not SSA

NO

YES

NO (e.g., opinion or conceptual)

**TO BE DECIDED**

UNSURE

Wrong study type – **EXCLUDE**

Label: wrong study type

YES

**Step 6: Is this empirical study or systematic review?**

- RCT, quasi-experimental, cohort, cross-sectional?

YES

NO

Wrong period – **EXCLUDE**

Label: before MDGs

**Step 5: Was the study conducted within MDGs?**

- Study conducted since 1998?
- Published since 2000?

**INCLUDE**
