# Supplementary material for: Implementation of medicines pricing policies in sub-Saharan Africa: systematic review
Source: Syst Rev. 2022 Dec 1;11:257. doi: 10.1186/s13643-022-02114-z (PMC9714131; doi:10.1186/s13643-022-02114-z)
Supplement: Supplementary file 3 — Additional file 3. Quality assessment of included studies. [file 13643_2022_2114_MOESM3_ESM.docx]

**Additional file 3:** Quality assessment of included studies

|  | **Quality appraisal checklist for analytical cross-sectional studies** | | | | | | | | | | | | | | | | | | | | | | | | | | | | | | | | |
| --- | --- | --- | --- | --- | --- | --- | --- | --- | --- | --- | --- | --- | --- | --- | --- | --- | --- | --- | --- | --- | --- | --- | --- | --- | --- | --- | --- | --- | --- | --- | --- | --- | --- |
|  | Quality appraisal checklist for analytical cross-sectional studies | Sabot, 2009 | Smith 2011 | Honda 2013 | Maı̈ga 2003 | Walwyn 2018 | Ali, 2012 | Ye, 2015 | Tougher, 2014 | | Bangalee, 2019 | Wiedenmaye 2019 | Cassar 2019 | Liu, 2017 | Maïga,7 Williams 2010 | Maiga 2010 | Moodley & Suleman 2019 | | Moodley & Suleman F 2019 | | Nicolosi & Gray 2009 | | Fink, 2014 | | Ongarora, 2019 | | Ponsar, 2011 | | Rothberg 2004 | | Guimier 2005 | | Cohen, 2013 |
| 1 | Were the criteria for inclusion in the sample clearly defined? | Y | Y | Y | Y | Y | Y | Y | Y | | Y | U | Y | Y | Y | Y | Y | | Y | | Y | | Y | | Y | | Y | | Y | | Y | | Y |
| 2 | Were the study subjects and the setting described in detail? | Y | Y | Y | Y | Y | Y | Y | Y | | Y | Y | Y | Y | Y | Y | Y | | Y | | Y | | Y | | Y | | Y | | Y | | Y | | Y |
| 3 | Was the exposure measured in a valid and reliable way? | Y | U | Y | Y | Y | Y | Y | Y | | Y | Y | Y | NA | Y | Y | Y | | Y | | U | | Y | | Y | | Y | | Y | | U | | Y |
| 4 | Were objective, standard criteria used for measurement of the condition? | Y | Y | Y | Y | Y | Y | Y | NA | | Y | U | Y | NA | Y | Y | Y | | Y | | U | | NA | | Y | | NA | | U | | Y | | Y |
| 5 | Were confounding factors identified? | N | Y | Y | Y | N | N | Y | NA | | N | N | N | NA | N | U | Y | | Y | | N | | U | | U | | N | | N | | U | | U |
| 6 | Were strategies to deal with confounding factors stated? | NA | NA | U | Y | N | NA | Y | NA | | NA | NA | NA | NA | NA | U | Y | | Y | | N | | U | | NA | | NA | | NA | | NA | | U |
| 7 | Were the outcomes measured in a valid and reliable way? | Y | Y | Y | Y | Y | Y | Y | Y | | Y | Y | Y | Y | Y | Y | Y | | Y | | Y | | Y | | Y | | Y | | Y | | U | | Y |
| 8 | Was appropriate statistical analysis used? | Y | Y | Y | Y | Y | Y | Y | Y | | Y | Y | Y | Y | Y | Y | Y | | Y | | Y | | Y | | Y | | Y | | Y | | Y | | Y |
|  | **Quality appraisal checklist for qualitative studies** | | | | | | | | | | | | | | | | | | | | | | | | | | | | | | | | |
|  | **Quality appraisal checklist for qualitative studies** | Ashigbie, 2016 | d'Almeida 2011 | Ali, 2009 | Walwyn 2018 | Wilson, 2012 |  |  | |  |  |  |  |  |  |  | |  | |  | |  | |  |  |  | |  | |  | |  | |
| 1 | Is there congruity between the stated philosophical perspective and the research methodology? | N | N | N | Y | N |  |  | |  |  |  |  |  |  |  | |  | |  | |  | |  |  |  | |  | |  | |  | |
| 2 | Is there congruity between the research methodology and the research question or objectives? | Y | U | Y | Y | Y |  |  | |  |  |  |  |  |  |  | |  | |  | |  | |  |  |  | |  | |  | |  | |
| 3 | Is there congruity between the research methodology and the methods used to collect data? | Y | U | Y | Y | Y |  |  | |  |  |  |  |  |  |  | |  | |  | |  | |  |  |  | |  | |  | |  | |
| 4 | Is there congruity between the research methodology and the representation and analysis of data? | Y | U | Y | Y | Y |  |  | |  |  |  |  |  |  |  | |  | |  | |  | |  |  |  | |  | |  | |  | |
| 5 | Is there congruity between the research methodology and the interpretation of results? | Y | Y | Y | Y | Y |  |  | |  |  |  |  |  |  |  | |  | |  | |  | |  |  |  | |  | |  | |  | |
| 6 | Is there a statement locating the researcher culturally or theoretically? | N | N | Y | N | N |  |  | |  |  |  |  |  |  |  | |  | |  | |  | |  |  |  | |  | |  | |  | |
| 7 | Is the influence of the researcher on the research, and vice- versa, addressed? | N | U | U | N | U |  |  | |  |  |  |  |  |  |  | |  | |  | |  | |  |  |  | |  | |  | |  | |
| 8 | Are participants, and their voices, adequately represented? | Y | U | N | Y | U |  |  | |  |  |  |  |  |  |  | |  | |  | |  | |  |  |  | |  | |  | |  | |
| 9 | Is the research ethical according to current criteria or, for recent studies, and is there evidence of ethical approval by an appropriate body? | Y | N | Y | Y | N |  |  | |  |  |  |  |  |  |  | |  | |  | |  | |  |  |  | |  | |  | |  | |
| 10 | Do the conclusions drawn in the research report flow from the analysis, or interpretation, of the data? | Y | Y | Y | Y | Y |  |  | |  |  |  |  |  |  |  | |  | |  | |  | |  |  |  | |  | |  | |  | |
|  | **Quality appraisal checklist for cohort studies** | | | | | | | | | | | | | | | | | | | | | | | | | | | | | | | | |
|  | **Quality appraisal checklist for cohort studies** | Steyn 2007 | de Jager, 2019 | Tran 2020 |  |  |  |  | |  |  |  |  |  |  |  | |  | |  | |  | |  |  |  | |  | |  | |  | |
| 1 | Were the two groups similar and recruited from the same population? | U | Y | Y |  |  |  |  | |  |  |  |  |  |  |  | |  | |  | |  | |  |  |  | |  | |  | |  | |
| 2 | Were the exposures measured similarly to assign people to both exposed and unexposed groups? | NA | NA | NA |  |  |  |  | |  |  |  |  |  |  |  | |  | |  | |  | |  |  |  | |  | |  | |  | |
| 3 | Was the exposure measured in a valid and reliable way? | NA | NA | NA |  |  |  |  | |  |  |  |  |  |  |  | |  | |  | |  | |  |  |  | |  | |  | |  | |
| 4 | Were confounding factors identified? | N | N | N |  |  |  |  | |  |  |  |  |  |  |  | |  | |  | |  | |  |  |  | |  | |  | |  | |
| 5 | Were strategies to deal with confounding factors stated? | NA | NA | NA |  |  |  |  | |  |  |  |  |  |  |  | |  | |  | |  | |  |  |  | |  | |  | |  | |
| 6 | Were the groups/participants free of the outcome at the start of the study (or at the moment of exposure)? | NA | NA | NA |  |  |  |  | |  |  |  |  |  |  |  | |  | |  | |  | |  |  |  | |  | |  | |  | |
| 7 | Were the outcomes measured in a valid and reliable way? | Y | Y | Y |  |  |  |  | |  |  |  |  |  |  |  | |  | |  | |  | |  |  |  | |  | |  | |  | |
| 8 | Was the follow up time reported and sufficient to be long enough for outcomes to occur? | NA | Y | U |  |  |  |  | |  |  |  |  |  |  |  | |  | |  | |  | |  |  |  | |  | |  | |  | |
| 9 | Was follow up complete, and if not, were the reasons to loss to follow up described and explored? | NA | Y | U |  |  |  |  | |  |  |  |  |  |  |  | |  | |  | |  | |  |  |  | |  | |  | |  | |
| 10 | Were strategies to address incomplete follow up utilized? | NA | U | U |  |  |  |  | |  |  |  |  |  |  |  | |  | |  | |  | |  |  |  | |  | |  | |  | |
| 11 | Was appropriate statistical analysis used? | Y | Y | Y |  |  |  |  | |  |  |  |  |  |  |  | |  | |  | |  | |  |  |  | |  | |  | |  | |

**Note:** Y(Yes), N(No), NA (Not Applicable) and U (Unclear)
